# Two MarR-Type Repressors Balance Precursor Uptake and Glycine Betaine Synthesis in *Bacillus subtilis* to Provide Cytoprotection Against Sustained Osmotic Stress

**DOI:** 10.3389/fmicb.2020.01700

**Published:** 2020-07-23

**Authors:** Bianca Warmbold, Stefanie Ronzheimer, Sven-Andreas Freibert, Andreas Seubert, Tamara Hoffmann, Erhard Bremer

**Affiliations:** ^1^Laboratory for Microbiology, Department of Biology, Philipps-University Marburg, Marburg, Germany; ^2^Department of Medicine, Institute for Cytobiology and Cytopathology, Philipps-University Marburg, Marburg, Germany; ^3^Faculty of Chemistry, Analytical Chemistry, Philipps-University Marburg, Marburg, Germany; ^4^Center for Synthetic Microbiology (SYNMIKRO), Philipps-University Marburg, Marburg, Germany

**Keywords:** osmotic stress, gene regulation, MarR-type repressors, DNA-binding, operators, gene duplication, *Bacillus subtilis*

## Abstract

*Bacillus subtilis* adjusts to high osmolarity surroundings through the amassing of compatible solutes. It synthesizes the compatible solute glycine betaine from prior imported choline and scavenges many pre-formed osmostress protectants, including glycine betaine, from environmental sources. Choline is imported through the substrate-restricted ABC transporter OpuB and the closely related, but promiscuous, OpuC system, followed by its GbsAB-mediated oxidation to glycine betaine. We have investigated the impact of two MarR-type regulators, GbsR and OpcR, on *gbsAB*, *opuB*, and *opuC* expression. Judging by the position of the previously identified OpcR operator in the regulatory regions of *opuB* and *opuC* [Lee et al. (2013) Microbiology 159, 2087−2096], and that of the GbsR operator identified in the current study, we found that the closely related GbsR and OpcR repressors use different molecular mechanisms to control transcription. OpcR functions by sterically hindering access of RNA-polymerase to the *opuB* and *opuC* promoters, while GbsR operates through a roadblock mechanism to control *gbsAB* and *opuB* transcription. Loss of GbsR or OpcR de-represses *opuB* and *opuC* transcription, respectively. With respect to the osmotic control of *opuB* and *opuC* expression, we found that this environmental cue operates independently of the OpcR and GbsR regulators. When assessed over a wide range of salinities, *opuB* and *opuC* exhibit a surprisingly different transcriptional profile. Expression of *opuB* increases monotonously in response to incrementally increase in salinity, while *opuC* transcription levels decrease after an initial up-regulation at moderate salinities. Transcription of the *gbsR* and *opcR* regulatory genes is up-regulated in response to salt stress, and is also affected through auto-regulatory processes. The *opuB* and *opuC* operons have evolved through a gene duplication event. However, evolution has shaped their mode of genetic regulation, their osmotic-stress dependent transcriptional profile, and the substrate specificity of the OpuB and OpuC ABC transporters in a distinctive fashion.

## Introduction

The soil dwelling Gram-positive bacterium *Bacillus subtilis* is frequently exposed to fluctuations in the environmental osmolarity, a process caused by flooding and drying (Mandic-Mulec et al., 2015; Hoffmann and Bremer, 2016). Desiccation of the soil restricts water-availability and thus raises the osmolarity of the surroundings relative to that of the cells’ interior (Stevenson et al., 2015). Consequently, water will flow out of the cell through the semi-permeable cytoplasmic membrane, thereby causing dehydration of the cytoplasm, a concomitant increase in molecular crowding, and a drop in turgor to physiologically unsuitable values (Wood, 2011; de Lima Alves et al., 2015; van den Berg et al., 2017; Bremer and Krämer, 2019).

Like many other microorganisms (Kempf and Bremer, 1998; Roeßler and Müller, 2001; Gunde-Cimerman et al., 2018), *B. subtilis* accumulates osmostress-relieving organic osmolytes, the compatible solutes. This counteracts water efflux and thereby prevents a drop in turgor, when *B. subtilis* faces hyperosmotic conditions (Hoffmann and Bremer, 2016, 2017). Accumulation of compatible solutes also optimizes the solvent properties and ionic composition of the cytoplasm as it prevents the build-up of a long-lasting high ionic strength cytoplasm. Such ionic unfavorable conditions of the cells’ interior would otherwise result from the massive uptake of potassium, the initial stress reaction of the cell when it faces high osmolarity surroundings (Dinnbier et al., 1988; Whatmore et al., 1990; Wood, 2011; Bremer and Krämer, 2019). The amassing of compatible solutes also protects the native structure of proteins and cellular sub-structures, and thus preserves the functionality of key biochemical reactions (Record et al., 1998; Barth et al., 2000; Bourot et al., 2000; Bolen and Baskakov, 2001; Ignatova and Gierasch, 2006; Wood, 2011; de Lima Alves et al., 2015; Stadmiller et al., 2017; Bremer and Krämer, 2019).

Compatible solutes are a restricted but chemically diverse group of highly water-soluble organic osmolytes (da Costa et al., 1998; Kempf and Bremer, 1998). *Bacillus subtilis* accumulates these stress-relieving and growth promoting compounds in a finely tuned process that is tightly linked to the degree of the osmotic stress imposed onto the cell (Brill et al., 2011; Hoffmann et al., 2013; Hoffmann and Bremer, 2016, 2017). For energetic reasons, import of compatible solutes is generally preferred over their synthesis (Oren, 1999). Accordingly, osmotically stressed *B. subtilis* cells can scavenge a great variety of pre-formed compatible solutes from environmental sources to achieve cytoprotection. It is thus able to sustain growth under osmotically unfavorable conditions (Hoffmann and Bremer, 2016, 2017). However, the ability of *B. subtilis* to synthesize compatible solutes is limited because only L-proline can be produced *de novo* (Whatmore et al., 1990; Brill et al., 2011; Hoffmann et al., 2017). In contrast, the synthesis of glycine betaine requires the prior import of the precursor choline (Boch et al., 1994, 1996; Kappes et al., 1999). None of the many other osmostress protectants used by *B. subtilis* can be synthesized and their accumulation thus requires the activities of osmotically controlled high-affinity import systems, the Opu family of transporters (Hoffmann and Bremer, 2017).

**FIGURE 1:**
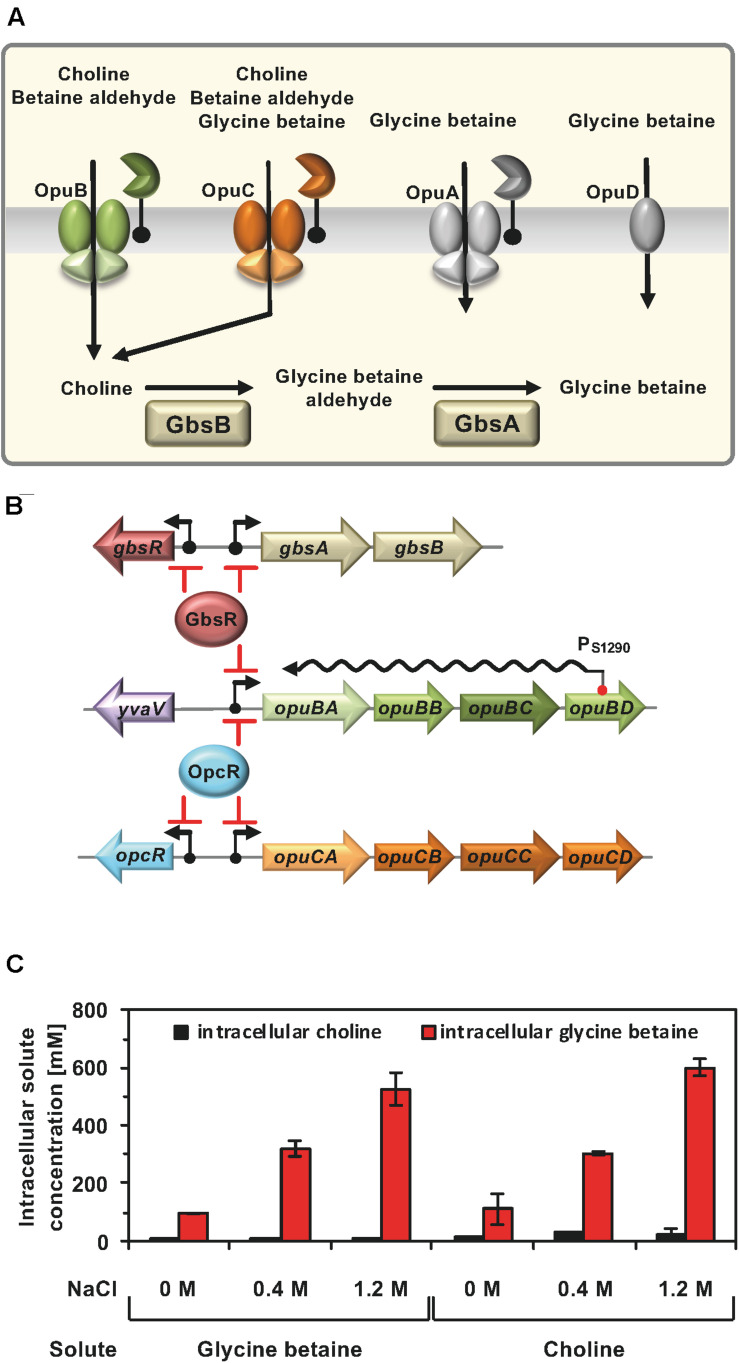
**(A)** Osmoprotectant uptake systems of *B. subtilis* and the choline to glycine betaine synthesis pathway. Depicted are four Opu transporters operating in *B. subtilis* along with some of their substrates. In addition, the import of choline and its enzymatic conversion to glycine betaine via the GbsB and GbsA dehydrogenases is shown. These data were taken from the literature (Hoffmann and Bremer, 2016, 2017). **(B)** Transcription of the *gbsAB* glycine betaine synthesis genes and of the *opuB* operon, encoding the choline-specific uptake system OpuB, is under control of the choline-responsive repressor GbsR (Nau-Wagner et al., 2012) and that of the *opuB* and *opuC* gene clusters is repressed by the OpcR protein (Lee et al., 2013). The GbsR and OpcR repressors also target the expression of their own structural genes (this study). Bent arrows mark the SigA-type promoters of the corresponding genes. *opuB* expression is also regulated by a long non-coding anti-sense RNA (S1290) which retards the full induction of this operon upon imposition a suddenly administered salt-shock. S1290 is expressed from a SigB-type promoter residing in *opuBD* and covers almost the entire length of the *opuB* sense-mRNA (Nicolas et al., 2012; Rath et al., 2020). **(C)** Cellular pools of glycine betaine and choline in osmotically stressed cells. The *B. subtilis* strain JH642 was grown in SMM with the indicated NaCl concentration, in the presence of either glycine betaine or choline (1 mM final concentrations). The glycine betaine and choline content of these cells were measured by LC-ESI-MS. The data given are the mean and standard deviations of two independent biological replicates in which each sample was assayed twice.

To deepen our understanding of the regulatory circuits and environmental cues contributing to the setting the compatible solute pool(s) to physiologically adequate levels (Hoffmann et al., 2013), we focus here on the systems that permit the import of choline via the OpuB and OpuC transporters (Kappes et al., 1999), its subsequent oxidation to glycine betaine by the GbsAB enzymes (Boch et al., 1996), and the scavenging of osmostress protectants via the promiscuous OpuC system (Hoffmann and Bremer, 2017; Teichmann et al., 2017; Figure 1A).

OpuB and OpuC are binding-protein-dependent ABC transporters, and both mediate high-affinity choline import under osmotic stress conditions (Kappes et al., 1999; Teichmann et al., 2017). However, choline is not an osmostress protectant *per se*; rather intracellular choline has to be enzymatically converted into glycine betaine. This involves a two-step oxidation process mediated by the GbsAB biosynthetic enzymes, a type-III alcohol dehydrogenase (GbsB) and a glycine betaine aldehyde dehydrogenase (GbsA) (Boch et al., 1994, 1996; Figure 1A). While OpuB possesses a restricted substrate profile (primarily for choline) (Teichmann et al., 2017), OpuC can import at least 15 chemically related osmostress protectants (Hoffmann and Bremer, 2011, 2017). This difference in substrate profile is quite remarkable because the operons encoding the *B. subtilis* OpuB and OpuC ABC transporters have evolved through a gene duplication event (Kunst et al., 1997; Kappes et al., 1999).

The OpuB and OpuC transporters differ not only in their substrate profile but also with respect to the regulation of their structural genes. A common denominator of *opuB* and *opuC* expression is the osmotic stimulation of their promoter activity (Kappes et al., 1999; Steil et al., 2003; Hahne et al., 2010; Nicolas et al., 2012; Lee et al., 2013). However, the molecular underpinnings of the required signal perception and transduction mechanism(s) are not well understood (Hoffmann and Bremer, 2016). The expression of the *opuB* operon and that encoding the GbsAB glycine betaine biosynthetic enzymes are controlled by the GbsR repressor (Figure 1B; Nau-Wagner et al., 2012), a member of the MarR-type superfamily of transcriptional regulators (Grove, 2013, 2017; Ronzheimer et al., 2018). Both the precursor (choline) and the intermediate in glycine betaine synthesis (glycine betaine aldehyde), (Figure 1A) (and their arsenic homologs, Hoffmann et al., 2018), serve as inducers of GbsR to relieve its DNA-binding activity. Newly synthesized, or imported, glycine betaine functions as an anti-inducer of GbsR, thereby preventing a wastefull over-accumulation of this metabolically inert stress protectant via uncontrolled synthesis (Nau-Wagner et al., 2012; Hoffmann et al., 2013, 2018). Notably, GbsR does not regulate the transcription of the *opuC* operon (Figure 1B), despite that the OpuC transporter can also serve as uptake system for choline (Figure 1A). Expression of the *opuC* operon, and simultaneously also that of *opuB*, is regulated by the GbsR-related OpcR repressor (Lee et al., 2013). However, OpcR has no influence on the expression of the *gbsAB* glycine betaine synthesis operon (Nau-Wagner et al., 2012; Figure 1B). In contrast to GbsR, no physiologically relevant inducer for the OpcR repressor is known.

Osmotic induction of *opuB* and *opuC* expression is well recognized, as is the GbsR- and OpcR-mediated transcriptional control of the *gbsAB*, *opuB*, and *opuC* operons. However, the transcriptional profile of the *opuB* and *opuC* operons in response to the severity of sustained osmotic stress has not been studied in detail. Likewise, it is unknown how the GbsR and OpcR repressors modulate the *opuB* and *opuC* transcriptional profile under persistent osmotic stress and whether these closely related MarR-type regulators use the same molecular mechanism to control transcription of their target genes. Collectively, the data that we provide here to address these issues demonstrate that evolution has substantially varied the osmostress-responsive transcriptional profile of the *opuB* and *opuC* operons. In addition, our data on GbsR, in conjunction with those reported earlier by Lee et al. (2013) for OpcR, reveal for the first time that the GbsR and OpcR repressors act through distinct molecular mechanism to control transcription of their respective target genes. Furthermore, salt-stress induction of *gbsR* and *opcR* transcription and auto-regulatory processes of the GbsR and OpcR regulators contribute to the setting of the compatible solute pool in osmotically stressed *B. subtilis* cells.

## Results

### Pools of Glycine Betaine and Choline Under Osmotic Stress Conditions

Import of glycine betaine, or its biosynthetic precursor choline, provide similar degrees of osmostress protection for *B. subtilis* (Boch et al., 1994, 1996; Kappes et al., 1999). Consequently, the cellular pools derived from imported glycine betaine (Hoffmann et al., 2013) and those resulting from the import of choline and its subsequent GbsAB-dependent enzymatic conversion into glycine betaine should be similar. However, this has not been tested experimentally. To address this issue, we grew the *B. subtilis* wild-type strain JH642 in a chemically defined medium (SMM) with glucose as the carbon source at various salinities and in the presence of 1 mM of either choline or glycine betaine. Liquid Chromatography-Electrospray Interface-Mass Spectrometry (LC-ESI-MS) was then used to measure the cellular content of these compounds. Conversion of choline into glycine betaine occurred efficiently since only residual amounts of the precursor molecule were detectable in the osmotically stressed *B. subtilis* cells (Figure 1C), attesting to the overall efficiency of the GbsAB glycine betaine biosynthetic enzymes (Boch et al., 1996, 1997). We observed that the pool size of imported glycine betaine and that formed via synthesis from the precursor choline matched closely. Furthermore, the size of the glycine betaine pools formed under both conditions was dependent on the degree of the imposed osmotic stress (Figure 1C).

### Mutational Analysis and DNA-Binding Studies Define the GbsR Operator of the *gbsAB* Operon

Transcription of the genes (*gbsAB*) for the enzymes mediating the conversion of choline into glycine betaine and those for choline import via the OpuB ABC transporter (Figures 1A,B) is coordinated by the choline-sensing GbsR repressor protein (Nau-Wagner et al., 2012). Based upon DNA sequence gazing alone, Nau-Wagner et al. (2012) proposed a GbsR binding site positioned down-stream of the *gbsAB* transcriptional start site. In addition, the position of a DNA sequence-related GbsR operator that overlapped the *opuB* promoter was suggested as well in this study (Nau-Wagner et al., 2012). However, bioinformatics analysis by Leyn et al. (2013) assessing the transcriptional regulatory network of *B. subtilis* on a global scale challenged these predictions and suggested an alternative GbsR operator site (Leyn et al., 2013). Given the disagreement of our previous report (Nau-Wagner et al., 2012) with the predictions made by Leyn et al. (2013), we decided to reinvestigate this issue. Hence, we targeted the originally proposed GbsR-binding site in the *gbsAB* operon (Nau-Wagner et al., 2012) through a series of site-directed mutagenesis experiments using the activity of a *gbsA-treA* transcriptional reporter fusion as a read-out for *gbsAB* promoter activity. Collectively, these data showed that the originally proposed GbsR operator for the *gbsAB* gene cluster by Nau-Wagner et al. (2012) is not correct as none of the introduced mutations resulted in loss of GbsR-dependent repression of transcription (Supplementary Table S1).

**FIGURE 2:**
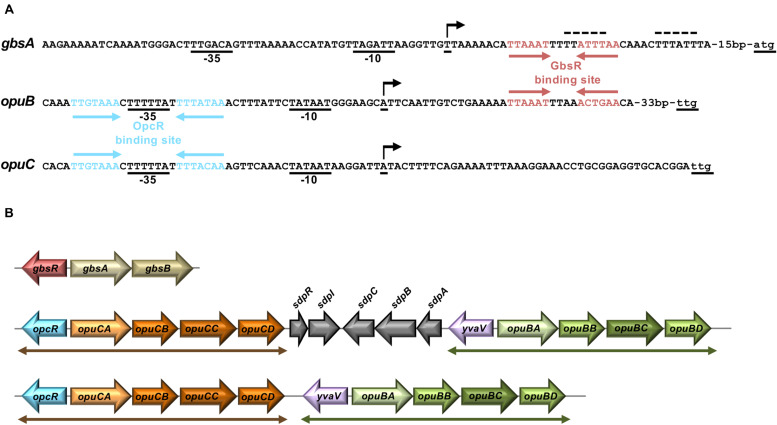
Genetic arrangement of *gbsAB*, *opuB*, and *opuC* operons and their promoter regions. **(A)** DNA sequence of the promoter regions of the *gbsAB*, *opuB*, and *opuC* operons and operator sites of the GbsR and OpcR repressors. Red arrows highlight the GbsR binding sites (this study); blue arrows mark the OpcR binding sites (Lee et al., 2013). The –35 and the –10 regions of the SigA-type promoters and the transcriptional start sites (indicated by a bent arrow) are highlighted. The transcriptional start site of the *gbsAB* and *opuB* mRNA has been experimentally determined through primer-extension analysis (Boch et al., 1996; Kappes et al., 1999), whereas that of the *opuC* operon is just predicted. The initially proposed GbsR binding site in the *gbsAB* regulatory region (Nau-Wagner et al., 2012) is marked by a dotted line. **(B)** The genetic structures of the *gbsAB*, *opuB*, and *opuC* operons and their divergently oriented *gbsR*-type regulatory genes in various members of the genus *Bacillus* is shown. In the genomes of *B. subtilis* 168 (Kunst et al., 1997) *B. subtilis* NCIB 3610 (Nye et al., 2017), and *Bacillus* sp. BS34A (Browne et al., 2015), the *opuB* and *opuC* gene clusters are separated by genes involved in the delay of sporulation and the onset of cannibalism (*sdpRI* and *sdpABC*) (Gonzalez-Pastor et al., 2003). In all other inspected genomes of members of the genus *Bacillus* (e.g., *Bacillus methylotrophicus*, *Bacillus mojavensis*, *Bacillus tequilensis*) that simultaneously possess *opuB* and *opuC* operons, the duplicated *opcR-opuC* and *yvaV-opuB* gene clusters (indicated by double-headed arrows) are juxtapositioned.

**FIGURE 3:**
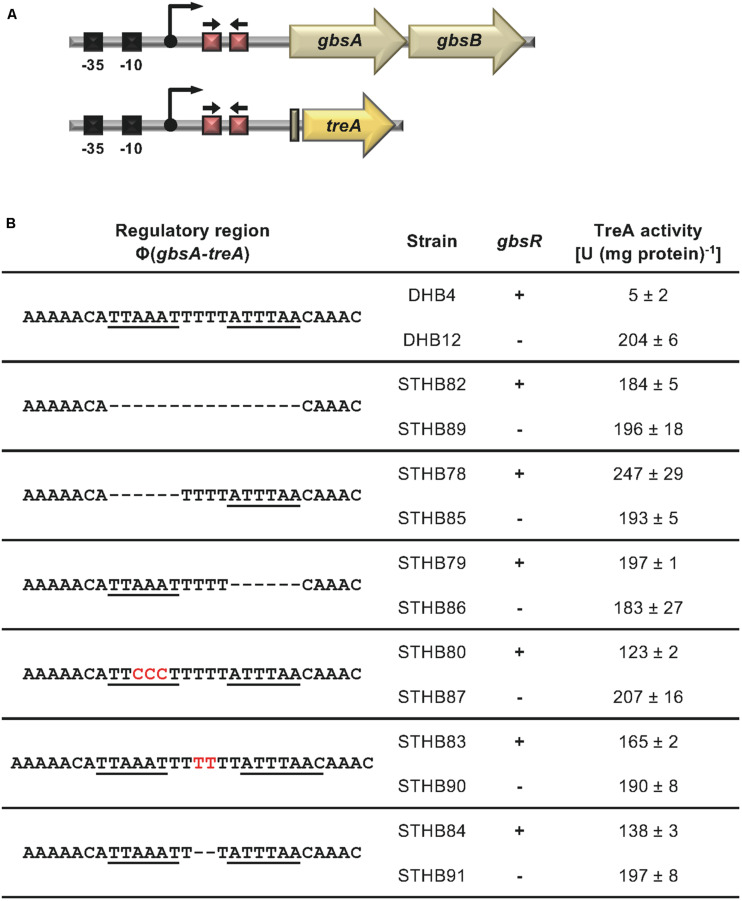
Mutational analysis of the GbsR binding site within the *gbsAB* regulatory region. **(A)** Schematic representation of the *gbsAB* promoter region. The –35 and –10 regions of the SigA-type promoter, the transcriptional start site (bent arrow) (Boch et al., 1996) and the suggested GbsR binding site (red boxes with divergently pointing arrows) are highlighted for both the wild-type *gbsAB* operon and the used *gbsA-treA* reporter fusion construct. **(B)** Transcriptional data of strains carrying *gbsA-treA* fusions with the indicated alterations of the putative GbsR binding site (underlined). Deletions are represented as dotted lines, and substitutions and insertions within the predicted GbsR binding site are highlighted in red. Cells were grown in SMM to early exponential growth phase (OD_578_ of 0.25) and were then exposed to high salinity (the final concentration of salt added to the cultures was 0.4 M NaCl) and 1 mM choline (final concentrations) to promote choline uptake and GbsR-mediated induction of reporter gene expression (when present in the strain) (Nau-Wagner et al., 2012). After further growth for 90 min, the cells were harvested by centrifugation for assays of the TreA reporter enzyme activity. The data given are the mean and standard deviations of two independent biological replicates, where each sample was assayed twice.

The position of the GbsR operator site predicted by Leyn et al. (2013) for the *gbsAB* operon is located downstream of the experimentally mapped transcription initiation site (Boch et al., 1996; Kappes et al., 1999). This predicted operator consists of an A/T-rich region with an inverted repeat, where the two half-sites are separated by four base pairs (Figure 2A). We subjected this region to a series of site-directed mutagenesis experiments that affected either the inverted repeats or their spacing. An isogenic pair of *B. subtilis* strains in which either the *gbsR* gene was intact or disrupted was then employed to study the effects of the operator-site mutations on the degree of GbsR-mediated repression using a chromosomal *gbsA-treA* operon fusion as a reporter system (Figure 3A). Loss of GbsR results in an approximately 40-fold de-repression of the transcriptional reporter fusion in an otherwise wild-type strain. All mutations introduced into the predicted GbsR operator sequence (Leyn et al., 2013) strongly de-repressed reporter fusion activity and resulted in the loss of GbsR responsiveness (Figure 3B).

**FIGURE 4:**
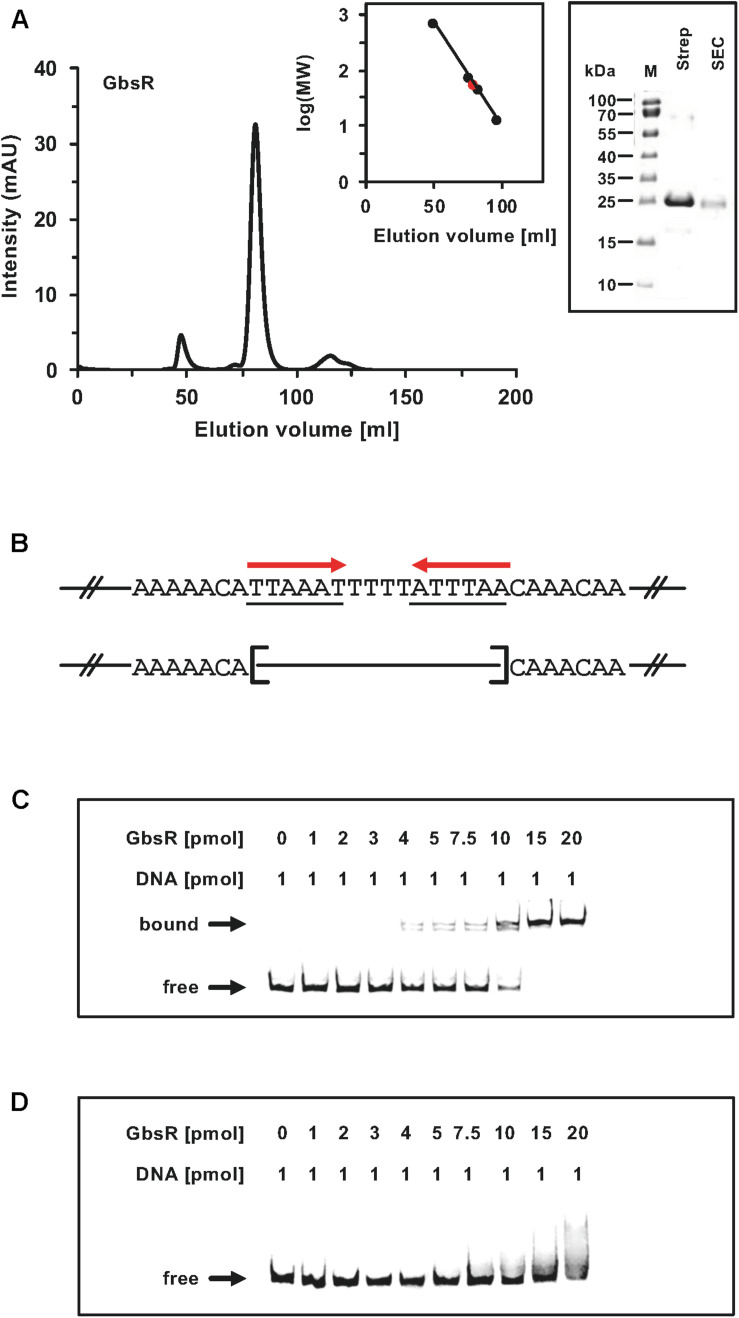
**(A)** Size-exclusion chromatography of the affinity purified GbsR-*Strep*-tag II recombinant protein. Immediately after the affinity purification of the heterologous produced GbsR-*Strep*-tag II protein, 2-ml protein solution (1 mg ml^–1^) was loaded onto a size-exclusion chromatography column (HiLoad 16/600 Superdex 200 pg) that was run in a buffer containing 100 mM potassium phosphate (pH 8) and 300 mM NaCl. Thyroglobulin (667 kDa), albumin (66 kDa), ovalbumin (43 kDa), and cytochrome C (12.4 kDa) were used to standardize the size-exclusion chromatography column. The purity and molecular mass of the GbsR-*Strep*-tag II recombinant protein was assessed by SDS-polyacrylamide gel electrophoresis (on a 15% poly-acrylamide gel); proteins were stained with InstantBlue^TM^ Sigma-Aldrich (Steinheim, Germany). **(B)** Illustration of the DNA fragments used for the DNA binding studies. Panels **(C,D)** show the interaction of the GbsR-*Strep*-tag II protein with the *gbsAB* promoter region. Increasing amounts of purified GbsR-*Strep*-tag II protein were incubated with fluorescently labeled [Dyomics 781 fluorescent dye (Microsynth AG, Balgach, Switzerland)] DNA fragments. The DNA fragments were electrophoretically separated in a native 8% polyacrylamide gel and the fluorescent label attached to the DNA fragments was detected using an Odyssey FC Imaging System (LI-COR Biosciences, Lincoln, NE, United States). **(C)** The *gbsA* DNA fragment used for the DNA-band shift assay contains the GbsR binding site while the *gbsA* DNA fragment used in panel **(D)** lacks.

Taken together, these genetic data strongly suggest that the inverted A/T-rich DNA-repeat found down-stream of the *gbsAB* transcriptional start site (Figure 2A) constitutes the GbsR operator. This was corroborated by a DNA-band-shift assay using DNA fragments containing or lacking the predicted GbsR operator site. For this experiment, we purified the dimeric GbsR protein to apparent homogeneity (Figure 4A). The purified protein bound the DNA fragment containing the predicted GbsR operator (Figures 4B,C) but a DNA fragment lacking it was not recognized (Figures 4B,D).

### Deletion and Mutagenesis of the GbsR Operator Does Not Prevent Osmotic Induction of *opuB*

**FIGURE 5:**
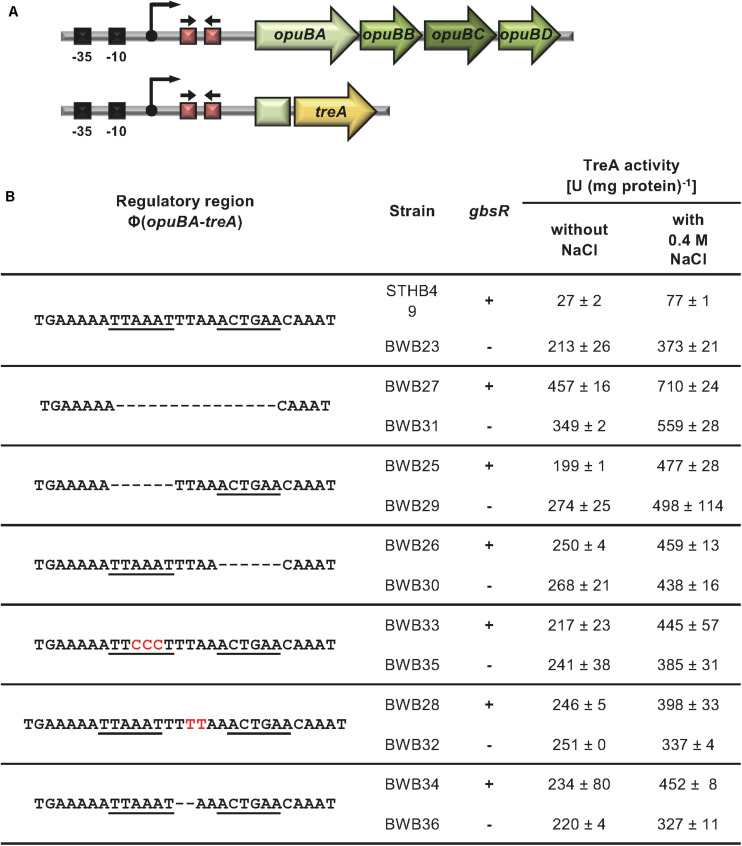
Mutational analysis of the GbsR binding site in the *opuB* regulatory region. **(A)** Schematic representation of the *opuB* promoter region. The –35 and –10 region of the SigA-type promoter, the transcriptional start site (bent arrow) (Kappes et al., 1999), and the suggested GbsR binding site (red boxes with divergently pointing arrows) are highlighted for both the wild-type *opuB* operon and the used *opuBA-treA* reporter fusion construct. **(B)** Analysis of strains carrying *opuBA-treA* fusions with the indicated alterations in the putative GbsR binding site (underlined). Deletions are represented as dotted lines, and substitutions and insertions within the predicted GbsR binding site are highlighted in red. Cells were grown in SMM to early exponential growth phase (OD_578_ of 0.25) when half of the cultures were subjected to osmotic up-shock elicited with NaCl. After further growth for 90 min, the cells were harvested by centrifugation for TreA reporter enzyme activity. The given data are the mean and standard deviations of two independent biological replicates, where each sample was assayed twice.

A previous study established that *opuB* expression is also under GbsR control (Nau-Wagner et al., 2012). We noted in the *opuB* regulatory region a putative GbsR operator similar in DNA sequence and spacing to that found in *gbsAB*. Like in gbsAB, this putative operator was also positioned down-stream of the transcription initiation site (Figure 2A). We targeted this DNA-sequence by site-directed mutagenesis. Transcriptional activity of the used *opuB-treA* reporter fusion (Figure 5A) increased by approximately eight-fold when GbsR was not present (Figure 5B). In all tested mutants with alterations in the putative GbsR operator sequence, basal activity of the *opuB-treA* reporter increased (between seven-fold and 17-fold) (Figure 5B). These data are fully consistent with the notation that the DNA-sequence highlighted in Figure 2A is the actual GbsR operator for the *opuB* operon.

Transcription of *opuB* is induced in response to high osmolarity (Kappes et al., 1999; Nau-Wagner et al., 2012). We therefore wondered whether loss of GbsR-responsiveness of *opuB* transcription would simultaneously alter osmotic control. Under mild osmotic stress (SMM containing additional 0.4 M NaCl), expression of the *opuB-treA* operon fusion is approximately three-fold induced (Figure 5B). In all strains carrying *opuB-treA* reporter fusions with altered GbsR operators, osmotic control was still retained (between 1.5 and 2.5-fold) when an intact *gbsR* gene was present (Figure 5B). When GbsR was absent, there was an about two-fold induction of *opuB-treA* reporter fusion activity upon the imposition of osmotic stress. Taken together, these data demonstrate that repression by GbsR and osmotic induction of *opuB* transcription are genetically separable events.

### Transcriptional Control of the *opuB* and *opuC* Operons Through GbsR-Type Regulators

*Bacillus subtilis* contains three GbsR-type regulatory proteins whose structural genes are positioned either adjacent to the *gbsAB* operon (*gbsR*), the *opuC* operon (*opcR/yvbF*), or the *opuB* operon (*yvaV*) (Nau-Wagner et al., 2012; Lee et al., 2013; Ronzheimer et al., 2018; Figure 2B). The GbsR/OpcR proteins posses a degree of amino acid sequence identity of 34%, that of the GbsR/YvaV pair is 35%, and the OpcR/YvaV proteins are 81% identical. In each case, the corresponding genes are oriented divergently from their neighboring *gbsAB*, *opuC*, and *opuB* operon (Figure 2B). The high amino acid sequence identity of the OpcR and YvaV proteins is a reflection of the duplication event that generated the *opuB* and *opuC* operons and flanking genes encoding the OpcR and YvaV GbsR-type regulatory proteins (Kunst et al., 1997; Kappes et al., 1999).

**FIGURE 6:**
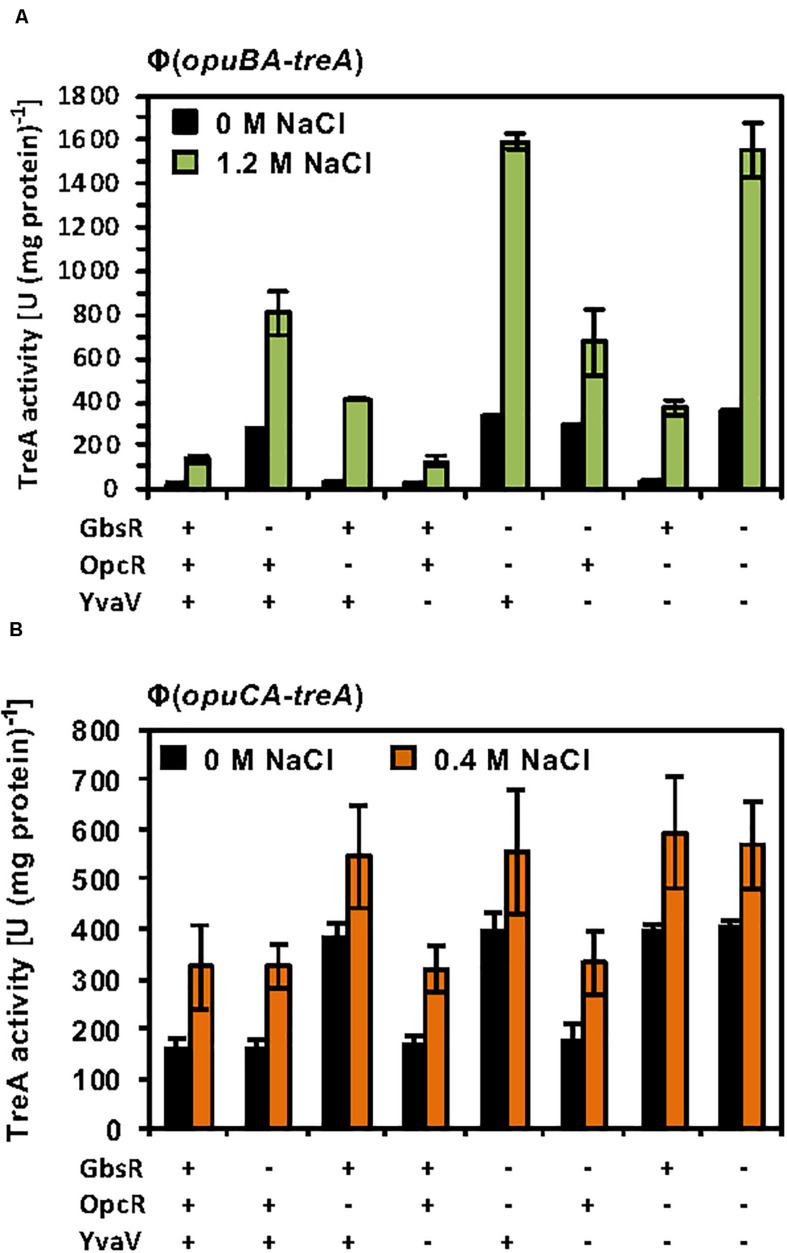
Response of *opuB and opuC* expression to the presence or absence of GbsR, OpcR and YvaV regulatory proteins. **(A)** *opuBA-treA* and **(B)** *opuCA-treA* reporter strains harboring the indicated gene disruption mutations were grown in SMM with the indicated NaCl concentrations until the cultures reached an OD_578_ of about 1–1.5. Cells were harvested by centrifugation and were subsequently assayed for their TreA reporter enzyme activity. The given data are the mean and standard deviations of four independent biological replicates, where each sample was assayed twice.

The considerable degree of amino acid sequence identity of the OpcR and YvaV proteins and the juxtaposition of the *yvaV* gene next to the *opuB* operon (Figure 2B) prompted us to explore a possible role of the YvaV protein in the control of *opuB* and *opuC* expression. To this end, we constructed a comprehensive set of *B. subtilis* strains to assess the individual contributions of the GbsR, OpcR, and YvaV regulatory proteins to *opuB* and *opuC* expression, or their combined effects. Each of these strains contained either a chromosomal *opuBA-treA* or an *opuCA-treA* reporter fusion as a read-out (Supplementary Table S2). In full agreement with data already reported by Lee et al. (2013), disruption of the *yvaV* gene by itself or in combination with either *gbsR* or *opcR* mutations had no noticeable effect on the transcriptional profile of the *opuB* and *opuC* reporter fusions, both in the presence and absence of osmotic stress (Figures 6A,B). We now found out that even in an *yvaV opcR gbsR* triple-mutant there was no noticeable effect of YvaV on either *opuB* or *opuC* (Figures 6A,B).

The GbsR and OpcR regulatory proteins jointly repress the activity of the *opuB* promoter (Nau-Wagner et al., 2012; Lee et al., 2013; Figure 6A). The position of the OpcR operator overlaps with the −35 region of the *opuB* and *opuC* promoters (Lee et al., 2013; Figure 2A) indicating that this repressor functions by preventing access of the RNA-polymerase to the promoter (Bervoets and Charlier, 2019). In contrast, our data (Figures 3B, 4C,D) show that GbsR targets a region down-stream of the *gbsAB* and *opuB* transcriptional start site (Figure 2A), suggesting that GbsR functions through a road-block mechanisms (Bervoets and Charlier, 2019). Under osmotic stress conditions, both the separate disruption of *gbsR* and *opcR* led to a partial de-repression of the *opuBA-treA* reporter fusion, genetic configurations under which the de-repressing effect of the loss of the GbsR repressor exceeded that of OpcR (Figure 6A). Consistent with the above outlined different modes of action of the OpcR and GbsR repressors, we found an additive effect of an *opcR gbsR* double mutation on *opuB* expression in *B. subtilis* cells grown under osmotic stress conditions (Figure 6A).

### Sustained Osmotic Stress Causes a Different Transcriptional Profile of the *opuB* and *opuC* Promoters

Previous studies demonstrated osmotic induction of *opuB* and *opuC* transcription both in response to sudden osmotic up-shocks triggered by the addition of NaCl to the growth medium and upon a sustained increase in salinity (Kappes et al., 1999; Steil et al., 2003; Hahne et al., 2010; Nicolas et al., 2012). We now found that enhanced expression of *opuB* and *opuC* under high salinity conditions is a reflection of a true osmotic cue, as it can be trigged by iso-osmotic solutions of both non-ionic (sucrose) and ionic (NaCl, KCl) osmolytes (Supplementary Table S3). As observed with other osmotically regulated *B. subtilis* genes (Hoffmann et al., 2013), osmotic induction of the *opuB* and *opuC* promoters requires the formation of an increased osmotic gradient across the cytoplasmic membrane. While both ionic and non-ionic osmolytes triggered enhanced expression of these operons, the addition of an osmotically corresponding concentration of glycerol, a membrane-permeable solute, to the growth medium did not induce the activities of the *opuB* or the *opuC* promoters (Supplementary Table S3).

**FIGURE 7:**
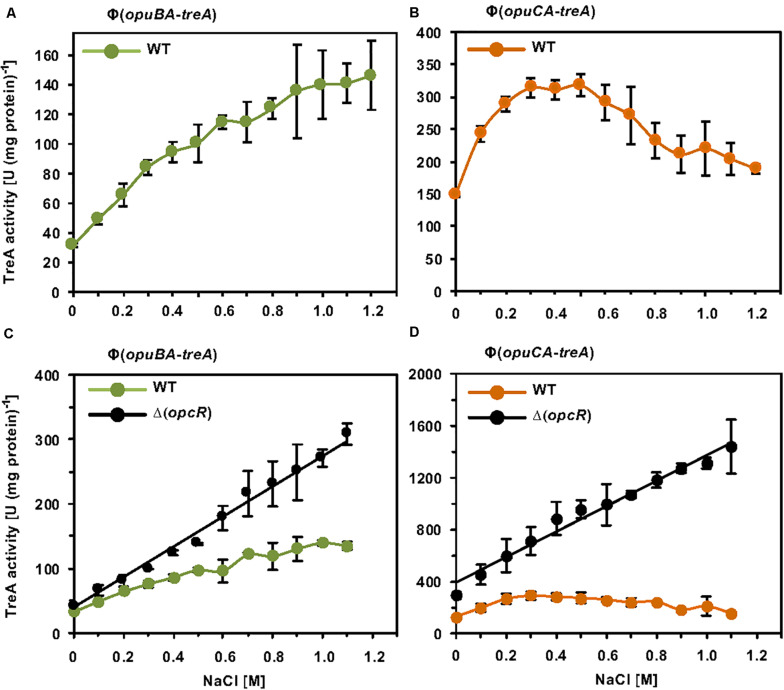
Osmotic control of *opuB* and *opuC* expression in response to extracellular salinities and the OpcR repressor. **(A)** The reporter fusion strains STHB49 (*opuBA-treA*) (green circles) and **(B)** STHB33 (*opuCA-treA*) (orange circles) were grown in SMM with increasing NaCl concentrations to mid-exponential growth phase (OD_578_ 1–1.5) and samples were subsequently assayed for TreA reporter enzyme activity. The given data are the mean and standard deviations of four independent biological replicates, which were each assayed twice. Panels **(C,D)** assess the influence of the OpcR repressor (Lee et al., 2013) on the expression of the reporter fusions. **(C)** The *opuBA-treA* reporter strains STHB49 (WT) (green circles) and STHB51 (Δ*opcR*) (black circles) and **(D)** the *opuCA-treA* reporter strains STHB33 (WT) (orange circles) and STH35 (Δ*opcR*) (black circles) were cultivated in SMM with the indicated NaCl concentrations until they reached mid-exponential growth phase (OD_578_ 1–1.5) and samples were then assayed for TreA reporter enzyme activity. The data given are the mean and standard deviations of two to four independent biological replicates, where each sample was assayed twice.

While these initial data assessing one particular osmotic stress condition on *opuB* and *opuC* promoter activity indicate that the transcriptional pattern of the *B. subtilis opuB* and *opuC* operons are similar, the tiling array study by Nicolas et al. (2012) suggests that both systems do not respond in the same manner to sustained osmotic stress (Nicolas et al., 2012). Since this issue has never been assessed at any level of detail, we systematically studied the transcriptional profile of *opuB* and *opuC* in cells adapted to different levels of osmotic stress. For this set of experiments, we grew the *opuB-treA* and *opuC-treA* reporter fusion strains in SMM with increasing salinities (0 M – 1.2 M NaCl) to the same mid-exponential growth phase (OD_578_ 1−1.5) and then assayed their TreA reporter enzyme activities. The *opuB* transcriptional profile showed in essence a continuous increase in promoter activity when the salinity of the medium was raised (Figure 7A). The transcriptional profile of *opuC* promoter activity, however, showed an entirely different pattern (Figure 7B). There was a steady increase in *opuC* promoter activity up to a moderate salinity of 0.3 M NaCl. Promoter activity then leveled off when the salinity was increased to 0.5 M NaCl, and even declined when the salinity of the medium was increased further up to 1.2 M NaCl (Figure 7B).

### Influence of OpcR on *opuB* and *opuC* Expression Under Sustained High Salinity Conditions

Because OpcR acts as a repressor for both the *opuB* and *opuC* promoters (Lee et al., 2013) (Figure 2A), we analyzed its influence on their salt-stress-responsive transcriptional profile. The absence of OpcR leads to a strong increase in promoter activity of both *opuB* and *opuC* over the entire range of the tested salinities (Figures 7C,D). Notably, in deviation of the *opuB* and *opuC* promoter activity in an *opcR* wild type strain (Figures 7A,B), loss of OpcR resulted in a transcriptional profile exhibiting a linear relationship with the degree of osmotic stress imposed onto the cells (Figures 7C,D). Therefore, we conclude that the OpcR repressor is required for the down-regulation of *opuB* and *opuC* promoter activities at higher levels of salinity, an effect that is particularly striking for *opuC*.

### OpcR Transcription Responses to Salt Stress and Is Auto-Regulated at High Salinity

**FIGURE 8:**
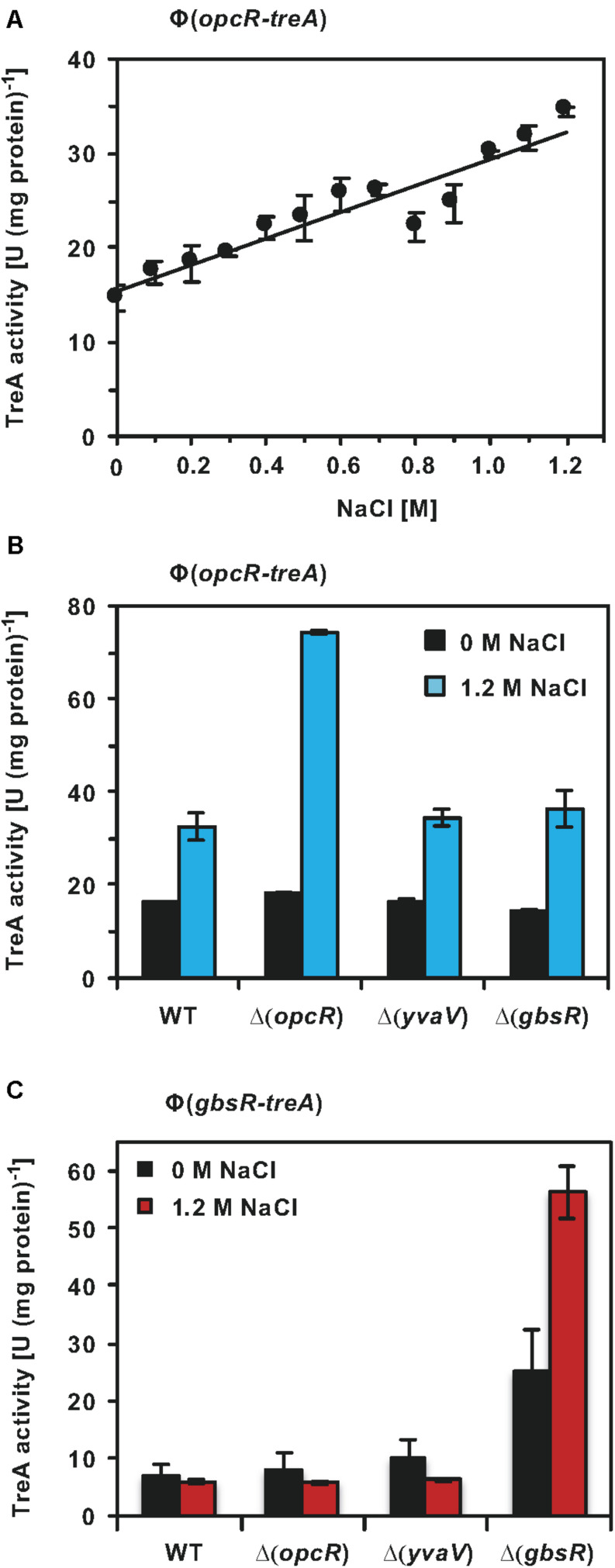
Regulation of *opcR* and *gbsR* transcription. **(A)** The *opcR-treA* reporter strain BWB127 was cultivated in SMM with increasing NaCl concentrations to mid-exponential growth phase (OD_578_ 1−1.5) and samples of the cultures were then assayed for TreA reporter enzyme activities. The given data are the mean and standard deviations of four independent biological replicates, where each sample was assayed twice. *opcR-treA* **(B)** and *gbsR-treA* **(C)** reporter strains of the wild-type strain and its mutant derivatives carrying gene disruption mutations affecting either *opcR*, *yvaV*, or *gbsR* were grown either in SMM or SMM with 1.2 M NaCl to an OD_578_ of 1−1.5. Samples were then assayed for TreA reporter enzyme activities. The given data are the mean and standard deviations of two independent biological replicates, where each sample was assayed twice.

Having established that OpcR modulates the transcriptional profiles of *opuB* and *opuC* under salt stress conditions (Figures 7C,D), we wondered whether the *opcR* promoter was responsive to salt stress as well. We investigated this issue using an *opcR-treA* reporter fusion integrated as a single copy in the chromosome of the wild-type strain. As expected for the transcriptional activity of a regulatory gene, the *opcR* promoter is rather weak; however, its activity responses to increasing salinity of the growth medium in a linear fashion (Figure 8A).

GbsR-type repressors belong to the super-family of MarR-type transcriptional regulators (Ronzheimer et al., 2018). Many MarR-type regulators control the expression of their own structural genes (Alekshun and Levy, 1997; Evans et al., 2001; Galan et al., 2003; Grove, 2013; Gupta et al., 2019). To assess if this is also true for OpcR and to further study a possible influence by the related regulatory proteins GbsR and YvaV on *opcR* promoter activity, we measured the activity of the *opcR-treA* reporter fusion in a set of strains with defects in the genes for these regulatory proteins. Disruption of *opcR* de-repressed the transcriptional activity of the *opcR-treA* reporter fusion by about two-fold under high-salinity conditions (Figure 8B). Therefore, OpcR acts as a repressor of its own structural gene when *B. subtilis* is grown under conditions of elevated salinity. However, no such effect was observed when the salinity of the growth medium was low (Figure 8B). Disruption of the *gbsR* and *yvaV* genes had no effect on *opcR* expression (Figure 8B).

### GbsR Auto-Regulates Its Structural Genes and gbsR Strongly Response to High Salinity When the GbsR Repressor Is Absent

We also tested whether the expression of *gbsR* was responsive to high salinity, its own gene product and to the GbsR-related OpcR and YvaV regulators. Using a chromosomal *gbsR-treA* reporter fusion, we found that OpcR and YvaV exerted no regulatory effect on *gbsR* transcription, while the loss of GbsR afforded an approximately 3.5-fold increase in expression of the fusion. Hence, *gbsR*, in contrast to *opcR*, is auto-regulated in the absence of salt stress (Figure 8C). The auto-regulatory effect of GbsR was particularly pronounced at high salinity. Comparing *gbsR-treA* expression in a wild-type strain and its isogenic *gbsR* mutant derivative in SMM containing 1.2 M NaCl, there was an almost ten-fold increase in *gbsR* transcription (Figure 8C).

### Viewing the Phylogenetic Distribution of the *yvaV-opuB*, *opcR-opuC* and *gbsR-gbsAB* Gene Clusters in a Genomic Context

**FIGURE 9:**
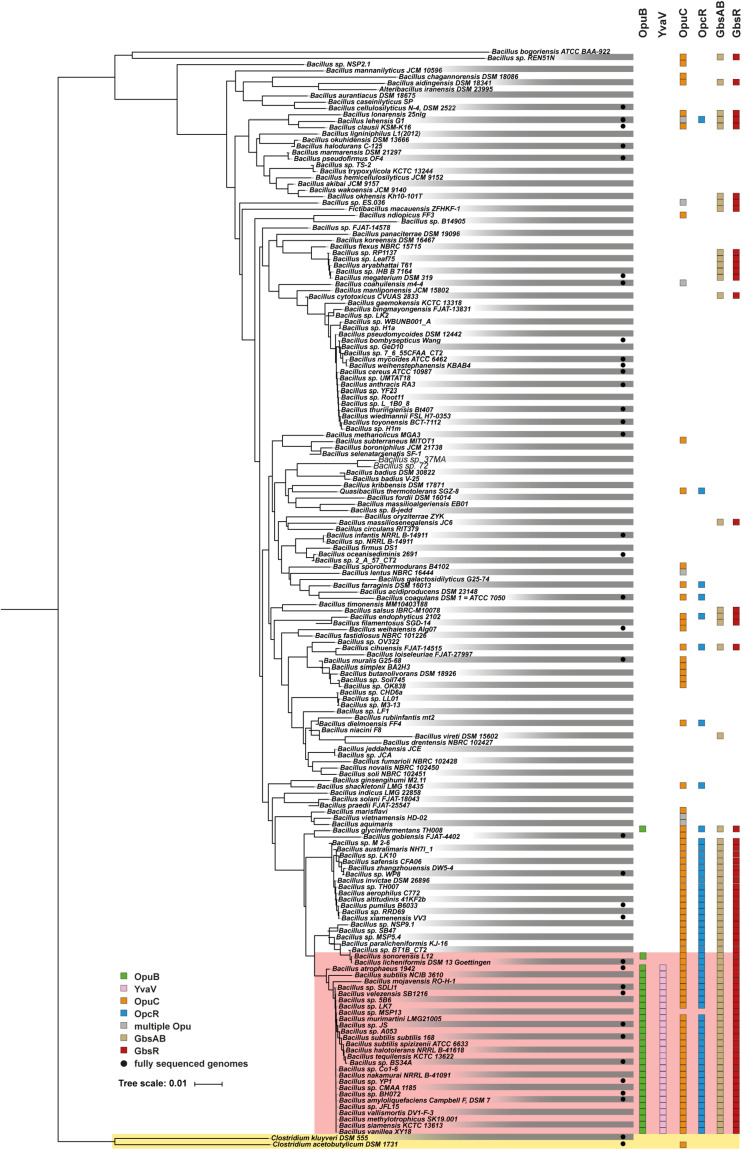
Distribution of the *opuB*, *opuC*, and *gbsAB* operons and of the corresponding repressor genes *gbsR*, *opcR*, and *yvaV* within members of the genus *Bacillus*. The phylogenetic tree of 173 members of the genus *Bacillus* whose 16S DNA sequences were deposited in the SILVA database (Glöckner et al., 2017), was adapted from a dataset originally reported by Teichmann et al. (2018). The color code is given in the figure. The red box covers the group of those Bacilli that simultaneously possess *yvaV-opuB* and *opcR-opuC* gene clusters. Two members of the genus *Clostridium* were used as the out-group in the construction of the 16S rRNA based phylogenetic tree. Data concerning the identity of the identified proteins are summarized in Supplementary Data Sheet 1.

The functionally different *opuB* and *opuC* operons of *B. subtilis* (Hoffmann and Bremer, 2017) have evolved in all likelihood through a gene duplication event (Kunst et al., 1997; Kappes et al., 1999). This is evidenced by the high degree of nucleotide sequence identities (72%) of the DNA segments encoding the *yvaV-opuB* and *opcR-opuC* gene clusters. Furthermore, the duplicated DNA segments are found in close proximity in the *B. subtilis* genome (Figure 2B). To analyze the phylogenomic distribution of the *yvaV-opuB* and *opcR-opuC* gene clusters and that of *gbsR-gbsAB*, we inspected the genome sequences of 173 *Bacillus* species for which 16S rRNA sequences were available for this group of genes (Figure 9). The types of proteins used for the BLAST-P analysis and the criteria used to assign OpuB and OpuC-type transporters and of the GbsAB glycine betaine biosynthetic enzymes are detailed in the section “Materials and Methods”. We limited our search to those *Bacillus* genome sequences that were available through the IMG/MER database as its genome browser readily allows the visualization of gene neighborhoods (Chen et al., 2019). We note that the genomes of the 173 *Bacillus* species used for our analysis comprise mostly draft sequences (Figure 9).

We found that *yvaV-opuB* and *opcR-opuC* gene clusters are only jointly present in a restricted sub-group of taxonomically closely related members of the genus *Bacillus* (Figure 9). This group of Bacilli include the legacy laboratory strains *B. subtilis* subspecies *subtilis* 168 (Kunst et al., 1997) and *B. subtilis* subspecies *spizizensis*, (Zeigler, 2011), as well as the *B. subtilis* strain 3610, an isolate widely used to study biofilm formation in this species (Branda et al., 2001). In all cases, where the *opuB* and *opuC* operons are jointly present, the duplicated chromosomal segment invariably also comprises the genes for the OpcR and YvaV GbsR-type regulatory proteins. However, the genes positioned between the *opuB* and *opuC* operons are not always the same. In a number of strains, including *B. subtilis*, the *yvaV-opuB* and *opcR-opuC* gene clusters are separated by fife genes involved in the delay of sporulation and the onset of cannibalism (*sdpRI* and *sdpABC*) (Gonzalez-Pastor et al., 2003; Figure 2B). In other inspected genomes (e.g., *Bacillus methylotrophicus*, *Bacillus mojavensis*, *Bacillus tequilensis*), they are positioned directly next to each other (Figure 2B). All representatives of those Bacilli with duplicated *yvaV-opuB* and *opcR-opuC* gene clusters also contained the *gbsAB* biosynthetic genes along with the *gbsR* regulatory gene (Figure 9).

The *opuB* operon is, with a single exception, not present outside of the group with the duplicated *yvaV-opuB* and *opcR-opuC* gene clusters, while *opuC* can be found in many other Bacillus species. In a group of Bacillus species taxonomically closely related to those possessing the *yvaV-opuB* and *opcR-opuC* gene clusters but lacking *opuB*, the *opcR-opuC* module is present along with that of the *gbsR-gbsAB* gene cluster (Figure 9). In all other inspected genome sequences of *Bacillus* species, *opuC*, *opcR*, and the *gbsR-gbsAB* module are more dispersedly present and *yvaV* is completely absent (Figure 9).

## Discussion

The GbsR and OpcR repressors coordinate, along with an environmental osmotic cue, the expression of operons encoding systems positioned at the core of the *B. subtilis* osmostress response, the accumulation of compatible solutes (Hoffmann and Bremer, 2016, 2017; Figures 1A,B). These two amino acid-sequence-related proteins (Nau-Wagner et al., 2012; Lee et al., 2013) belong to the same sub-family of MarR-type transcriptional regulators (Grove, 2017; Ronzheimer et al., 2018). However, their target gene profile (Figure 1B) and mode of gene repression are different. Judging by assessing the position of the operators for OpcR and GbsR relative to that of the promoters and transcriptional start-sites (Figure 2A), these two repressors act via different molecular mechanisms. OpcR acts by restricting access of RNA-polymerase to the −35 region of the *opuB* and *opuC* promoters (Lee et al., 2013), while GbsR must function via a roadblock mechanism as its operators are positioned downstream of the *gbsAB* and *opuB* transcription initiation sites (this study) (Figure 2A). These mechanistically different regulatory modes of repressors (Bervoets and Charlier, 2019) come both into play for the GbsR- and OpcR-mediated control of *opuB* expression (Figure 2A), where GbsR and OpcR repress transcription in an additive manner (Figure 6A).

The GbsR and OpcR operators are highly A/T-rich but they are not identical, neither in their DNA-sequences, nor in the length of their spacers (Figure 2A). The crystal structure of the DNA-binding protein Mj223 from *Methanococcus jannaschii* (PDB accession code: 1ku9) (Ray et al., 2003), a MarR-type protein of unknown function, can be used to establish *in silico* models for GbsR-type proteins (Nau-Wagner et al., 2012; Hoffmann et al., 2018; Ronzheimer et al., 2018). Mj223 is predicted to recognize its (unknown) operator sequence via a winged-helix-turn-helix DNA-binding motif (Ray et al., 2003; Grove, 2017). Such a motif is also present in the N-terminal domains of the GbsR and OpcR regulatory proteins, and in YvaV (Supplementary Figure S1). Hence, by inference, the dimeric GbsR and OpcR repressors should recognize their cognate operators in a fashion similar to other structurally and biochemically well studied MarR-type regulatory proteins (Grove, 2013, 2017) [see Supplementary Figure S2 for an extended amino acid sequence alignment of GbsR-, OpcR- and YvaV-type proteins in Bacilli]. However, the DNA sequence of the differently configured GbsR and OpcR operators, in particular with respect to the length of their spacers (Figure 2A), suggest that there must be notable differences in the way in which these closely related repressor proteins interact with their specific DNA targets via their N-terminal DNA-reading heads.

In a phylogenomic approach, we analyzed the distribution of the genes encoding the OpuB and OpuC transporters in members of the genus *Bacillus* along with that of the *gbsAB* glycine betaine biosynthesis genes (Figure 9). Building on this dataset of 173 genome sequences of Bacilli (Teichmann et al., 2018), we found that the DNA sequences of the GbsR and OpcR operators are highly conserved, both with respect to their actual DNA sequence, the length of the spacer, and their position relative to the promoter of the *gbsAB*, *opuB* and *opuC* operons (Supplementary Figures S3–S5). Hence, the data reported here and those published previously (Nau-Wagner et al., 2012; Lee et al., 2013) collectively suggest that the mechanistic different modes of actions of the *B. subtilis* GbsR and OpcR repressors to control gene expression (Bervoets and Charlier, 2019) are likely evolutionarily conserved for their orthologs present in many members of the genus *Bacillus*.

Previous studies revealed an osmotic transcriptional control of the *B. subtilis opuB* and *opuC* promoters. Moderate osmotic up-shocks trigger a rapidly enhanced transcription of both operons (Kappes et al., 1999; Hahne et al., 2010; Nau-Wagner et al., 2012; Nicolas et al., 2012; Lee et al., 2013). In contrast, continuous severe osmotic stress (elicited with 1.2 M NaCl) that requires a growth-restoring cellular adaptation of *B. subtilis* (Hoffmann and Bremer, 2016) results in a different transcriptional response of the *opuB* and *opuC* promoters (Figures 7A,B). It was observed in a previous study that, while *opuB* expression is strongly enhanced, *opuC* transcription was seemingly not induced by sustained high salinity (Nicolas et al., 2012). However, a shortcoming of the tiling array data reported by Nicolas et al. (2012) is that only a single salt concentration was used to assess changes in the transcriptome of continuously osmotically stressed *B. subtilis* cells.

In our study, we monitored the transcriptional profiles of *opuB* and *opuC* in much greater detail by assessing them over a wide spectrum of salinities (0 – 1.2 M NaCl) (Figures 7A,B). Accordingly, this approach provided a more complete picture of the *opuB* and *opuC* transcriptional response to persisting osmotic stress of different intensities. For the first time striking differences in the activity profile of the *opuB* and *opuC* promoters became apparent. While *opuB* promoter activity increased almost monotonously in response to concomitant increases in the salinity of the growth medium (Figure 7A), that of *opuC* increased only at moderate salt concentrations, leveled off when the salinity of the growth medium was incrementally increased, and then declined again at higher salinities (Figure 7B). Loss of OpcR caused a striking difference in the activity of the *opuB* and *opuC* promoters by making them linearly responsive to the degree of osmotic stress imposed onto the cell (Figures 7C,D). Hence, in a wild-type background OpcR seemingly functions to limit *opuB* and *opuC* expression, even under osmotic conditions that are challenging for the growth of *B. subtilis* (Boch et al., 1994).

The glycine betaine pool attained via import of a *B. subtilis* wild-type cell is linearly dependent on the degree of the imposed osmotic stress (Hoffmann et al., 2013). It is counter-intuitive, at least at first sight, that osmotically challenged cells down-regulate the expression of *opuC* (Figure 7B), an operon encoding the transporter with the broadest substrate profile of all compatible solute transporters operating in *B. subtilis* (Hoffmann and Bremer, 2016, 2017). However, in order to maintain a physiologically adequate osmoprotective glycine betaine pool (and for that matter of other compatible solutes as well), the uptake rates for glycine betaine and its biosynthetic precursor choline must be adjusted to both the level of the imposed osmotic stress (Hoffmann et al., 2013) but concomitantly also to the dynamic increase in cell volume prior to division of the *B. subtilis* cell (Dai and Zhu, 2018; Reyes-Lamothe and Sherratt, 2019).

Salinities higher than 0.5 M NaCl added to a minimal medium with glucose as the sole carbon and energy source cause a strong growth rate decrease of *B. subtilis* cultures; doubling times increase from 1 h to nearly 5 h (in SMM with added 1.2 M NaCl) (Boch et al., 1994; Hoffmann et al., 2013). Thus, it makes physiological sense that the uptake-rates of the cell for osmostress protectants must be reduced when the growth rate drops. This would prevent a possible detrimental over-accumulation of compatible solutes that could potentially increase turgor pressure to physiologically unsustainable values necessitating the opening of mechanosensitive channels (Wood, 2011; Bremer and Krämer, 2019). OpcR-mediated repression of *opuB* and *opuC* transcription under severe osmotic stress conditions might therefore be a useful strategy to achieve this. Likewise, the GbsR-mediated control of *opuB* and *gbsAB* expression, and its feed-back inhibition by intracellular glycine betaine pools (Nau-Wagner et al., 2012) also contributes to this physiologically relevant process. Embedded in this homeostatic system are not only the DNA-binding activities of the OpcR and GbsR repressors (Figure 1B) but also the cellular levels of OpcR and GbsR proteins as well. The transcriptional profiles of *opcR* and *gbsR* are notably different with respect to osmotic control and auto-regulation. While transcription of *opcR* is salt stress responsive in a wild-type background, osmoregulation of *gbsR* becomes only apparent when the GbsR repressor is non-functional (Figures 8A–C). Conversely, auto-regulation of *opcR* is only notable under osmotic stress conditions, while that of *gbsR* is apparent under non-stressed conditions and when the cells are challenged by high salinity (Figure 8C).

The gene duplication generating the *opuB* and *opuC* loci has occurred in a restricted and phylogenetically closely related group of *Bacillus* species (Figure 9). Along with the duplication of the genes for the OpuB and OpuC ABC transporters, the juxtapositioned *yvaV* and *opcR* genes were also duplicated (Figure 2B). While a regulatory function of the OpcR repressor for *opuB* and *opuC* expression has clearly been established (Lee et al., 2013, this study), no regulatory function of the amino acid sequence related YvaV protein (OpcR and YvaV: 81% sequence identity) has so far been discovered. It is neither involved in *opuB* and *opuC* expression (Figures 6A,B) nor required for the regulation of the *gbsAB* glycine betaine synthesis operon (Nau-Wagner et al., 2012). Loss of YvaV does also not affect the transcriptional profile of the *opcR* and *gbsR* genes when the other GbsR.-type repressors GbsR and OpcR are intact (Figures 8B,C). Because the *yvaV* gene is not transcriptionally silent (Nicolas et al., 2012), growth conditions await discovery where an effect of the YvaV MarR-type protein on gene expression become apparent.

Osmotically stimulated transcription of the *opuB* and *opuC* operons occurs under the control of SigA-type promoters (Kappes et al., 1999; Figure 2A) and osmostress responsiveness is not lost even in *opcR*, *gbsR*, *yvaV* triple mutants. Only the allover transcriptional levels of *opuB* and *opuC* are affected by loss of OpcR and GbsR (Figures 6A,B) and hence, the *opuB* and *opuC* promoters are able to response to an osmotic cue in the absence of these MarR-type regulators. It is unknown how the *B. subtilis* cell perceives osmotic stress, and genetically processes this information to stimulate *opuB* and *opuC* transcription (Hoffmann and Bremer, 2016).

The interplay of the GbsR and OpcR repressor proteins for *opuB* and *opuC* transcription (Nau-Wagner et al., 2012; Lee et al., 2013) and the compatible solute pools in osmotically stressed cells (Hoffmann et al., 2013) provides *B. subtilis* with an effective stress-relieving control unit. Combined, this module allows a finely tuned adjustment of *gbsAB*, *opuB*, and *opuC* transcription in response to both environmental (osmotic) and cellular (compatible solute pools) cues. While the inducers (choline and glycine betaine aldehyde, and their arsenic analogs) and effectors (glycine betaine and arsenobetaine) that dictate the DNA-binding properties of GbsR are known (Nau-Wagner et al., 2012; Hoffmann et al., 2018), corresponding information for the OpcR repressor is still lacking.

## Materials and Methods

### Chemicals

Antibiotics were purchased from Carl Roth (Karlsruhe, Germany), United States Biochemical Corp. (Cleveland, OH, United States), Sigma-Aldrich (Steinheim, Germany), and InvivoGen (San Diego, CA, United States). Choline, glycine betaine and the chromogenic substrate for the TreA enzyme assays, *para*-nitrophenyl-α-D-glucopyranoside (α-PNPG), were purchased from Sigma-Aldrich (Steinheim, Germany). Anhydrotetracycline-hydrochloride (AHT), Strep-Tactin Superflow chromatography material, and desthiobiotin were obtained from IBA GmbH (Göttingen, Germany). Marker proteins to standardize size-exclusion chromatography columns were purchased from GE Healthcare (München, Germany) and from Sigma-Aldrich (Steinheim, Germany), respectively.

### Bacterial Strains

All *B. subtilis* strains used in this study are derivatives of the domesticated laboratory strain JH642 (*trpC2 pheA1*) (Smith et al., 2014). Their genotypes are listed in Supplementary Table S2. The *Escherichia coli* strains DH5α (Invitrogen, Carlsbad, CA, United States) and Top10 (Invitrogen, Carlsbad, CA, United States) were used for the construction of plasmids and their routine maintenance. The *E. coli* B strain BL21 (Dubendorff and Studier, 1991) was used for overproduction of the recombinant GbsR protein.

### Media and Growth Conditions

Bacterial strains were propagated on Luria-Bertani (LB) agar plates or in liquid media at 37°C. *Bacillus subtilis* was grown in Spizizen’s minimal medium (SMM) with 0.5% glucose as a carbon source and a solution of trace elements (Harwood and Cutting, 1990). L-Tryptophan (20 mg l^–1^) and L-phenylalanine (18 mg l^–1^) were added to the medium to satisfy the auxotrophic needs of *B. subtilis* strain JH642 (*trpC2 pheA1*) (Smith et al., 2014) and its derivatives (Supplementary Table S2). The osmolarity of the medium was increased by the addition of NaCl (5 M stock solution), KCl (1.2 M stock solution), glycerol (10.5 M stock solution) or sucrose (2 M stock solution) to the final concentrations indicated in the individual experiments. Choline and glycine betaine were filter sterilized and added to the growth medium in the indicated concentrations from 100 mM stock solution. Liquid cultures of *B. subtilis* were grown at 37°C in 100-ml Erlenmeyer flasks (containing 20 ml of medium) in a shaking water bath (set to 220 rpm). Bacterial growth was monitored photometrically as the optical density of cultures at 578 nm (OD_578_). Cultures were inoculated to an OD_578_ of 0.1 from exponentially growing pre-cultures prepared in SMM.

### Construction of Plasmids and *Bacillus subtilis* Strains

Isolation of chromosomal DNA from *B. subtilis* and transformation of *B. subtilis* strains with PCR products, plasmids, or chromosomal DNA followed standard procedures (Harwood and Cutting, 1990). *Bacillus subtilis* mutants carrying chromosomal deletions of either the *gbsR*, *opcR*, or *yvaV* genes were constructed by transforming the *B. subtilis* strain JH642 and its derivatives (Supplementary Table S2) with long-flanking region PCR products (Kuwayama et al., 2002). The oligonucleotides used for the amplification of the 5′- and 3′-flanking regions of the desired gene and of the antibiotic resistance cassettes used to disrupt the coding region are listed in Supplementary Table S4. The spectinomycin resistance cassette inserted in the *gbsR* gene was amplified using plasmid pDG1726 (Guerout–Fleury et al., 1996) as the template), and the zeocin resistance cassette inserted in the *opcR* gene was derived from plasmid p7Z6 (Yan et al., 2008; Supplementary Table S5). The tetracycline resistance cassette used to disrupt the *yvaV* locus was amplified from plasmid pDG1515 (Guerout–Fleury et al., 1996; Supplementary Table S5).

To construct the *opuB–treA* transcriptional reporter fusion, a 1037–bp DNA fragment carrying the *opuB* promoter and part of the *opuBA* coding region (Kappes et al., 1999) was amplified by using primers containing artificial SmaI and BglII restriction sites at their 5′-ends (SmaI-opuB for and BglII-opuB; Supplementary Table S4). The resulting DNA fragment was then inserted into the vector pJMB1 carrying a promoterless *treA* reporter gene (Schöck et al., 1996; Hoffmann et al., 2013; Supplementary Table S5), which had been cut with SmaI and BglII; this yielded plasmid pSTH67 [*amyE::opuB-treA-cat::amyE* (Supplementary Table S5)]. Primers OpuC TreA1 for and OpuC TreA rev were used to amplify the 1014-bp DNA fragment used for the construction of the *opuC-treA* transcriptional reporter fusion, and primers OpcR-treA_for and OpcR-treA_rev (Supplementary Table S4) were employed for the amplification of the 1066-bp chromosomal DNA fragment used for the construction of the *opcR-treA* reporter fusion. The primers carried artificial SmaI and BamHI restriction sites at their 5′-ends, which allowed the cloning of the amplified DNA-segments into the vector pJMB1 (Hoffmann et al., 2013; Supplementary Table S5) that had been cut with SmaI and BamHI. These genetic manipulations yielded plasmids pSTH62 (*amyE::opuC-treA-cat::amyE*) and pBW34 [*amyE::opcR-treA-cat::amyE* (Supplementary Table S5)]. In a similar approach, we constructed a *gbsR-treA* fusion by inserting a 964-bp PCR fragment, covering part of *gbsR*, the promoter regions for the *gbsR* and *gbsAB* genes, and a section of *gbsA*, in front of the promoter-less *treA* reporter gene in plasmid pJMB1 (Hoffmann et al., 2013; Supplementary Table S5). This yielded pGNB10 (*amyE::gbsR-treA-cat::amyE*). The primers used for the amplification of the “gbsR-*gbsA-treA*” genomic segment were GbsR-TreA_for and GbsR-TreA_rev (Supplementary Table S4).

The various *treA* reporter fusion constructs were stably integrated as a single copy into the *amyE* gene of the *B. subtilis* chromosome by DNA transformation and a subsequent double homologous recombination event (Harwood and Cutting, 1990). The antibiotics chloramphenicol (5 μg ml^–1^), erythromycin (2 μg ml^–1^), kanamycin (5 μg ml^–1^), spectinomycin (100 μg ml^–1^), zeocin (35 μg ml^–1^), and tetracycline (10 μg ml^–1^) were used for the selection of gene disruption mutations in *B. subtilis* after transformation. The resulting strains are listed in Supplementary Table S2.

Mutations in the *B. subtilis gbsA* and *opuB* promoter regions were generated with the Q5 site-directed mutagenesis kit (New England Biolabs, Ipswich, United States) and a series of appropriate mutagenic primers (Supplementary Table S4). Plasmid pDH2 (Nau-Wagner et al., 2012) carrying the wild-type *gbsA* promoter region and plasmid pSTH67 carrying the wild-type *opuB* promoter region (Supplementary Table S5) served as templates for the site-directed mutagenesis experiments. For overproduction of the GbsR repressor (Nau-Wagner et al., 2012), the coding region of *gbsR* was amplified from chromosomal DNA of *B. subtilis* JH642 using primers gbsR_B.sub_IBA3_for and gbsR_B.sub_IBA3_rev (Supplementary Table S4), and the resulting DNA fragment was then inserted into the expression vector pASG-IBA3 (IBA, Göttingen, Germany) to obtain the GbsR overproduction plasmid pSTH02 (Supplementary Table S5). This construct is similar in its genetic structure to the previously described *gbsR* overexpression plasmid pDH1 (Nau-Wagner et al., 2012), but differs in the position of the *Strep*-tag II affinity peptide (SA-WSHPQFEK); it is now present at the C-terminal end of the GbsR protein. The *gbsR* gene present in plasmid pSTH02 is expressed form a *tet* promoter, a promoter whose activity is positioned under the control of the anhydrotetracyline (AHT)-inducible TetR repressor. The *tetR* gene is encoded by the backbone of the expression plasmid (IBA, Göttingen, Germany).

### Measurements of Intracellular Glycine Betaine and Choline Pools

To determine intracellular solute pools of glycine betaine and choline, the *B. subtilis* strain JH642 was grown in 50 ml of SMM, SMM with 0.4 M NaCl, or SMM with 1.2 M NaCl in the presence of 1 mM glycine betaine or 1 mM choline until the cultures reached an OD_578_ of about 2−2.5. The cells were harvested by centrifugation (2,400 × g) for 10 min at 37°C and were washed once with iso-osmotic pre-warmed growth medium (at 37°C) lacking the choline or glycine betaine osmostress protectants. Cell extracts were prepared using 80% (vol/vol) ethanol, and the supernatant was centrifuged (22,000 × *g*) to remove cellular debris. Choline and glycine betaine were detected and quantified using Liquid Chomatography-Electrospray Interface-Mass Spectrometry (LC-ESI-MS) as described previously (Hoffmann et al., 2018). The *B. subtilis* strain TMB118, which lacks the OpuA, OpuB, OpuC, and OpuD transporters (Teichmann et al., 2017; Supplementary Table S2), was used as a negative control to assess the true import of glycine betaine and choline. Extracts of this strain showed an average glycine betaine content of 20 mM and of 0.1 mM choline, respectively. These values represent the incomplete removal of glycine betaine or choline from the outside of intact cells during harvesting of the samples, since the mutant strain TMB118 cannot import glycine betaine or choline (Hoffmann et al., 2013). Therefore, these values were always subtracted from the pool sizes of these compounds observed in the wild-type *B. subtilis* strain JH642, a strain that possesses all compatible solute importers (Hoffmann et al., 2013; Hoffmann and Bremer, 2017). Values for the volume of *B. subtilis* cells were taken from Hoffmann et al. (2013) to express the glycine betaine and choline content of the cells in mM (Hoffmann et al., 2013).

### TreA Reporter Enzyme Activity Assays

Aliquots (1.8 ml) from cultures of *B. subtilis* strains carrying various *treA* reporter gene fusions (Schöck et al., 1996) as single-copy constructs (stably inserted into *amyE* via double-homologous recombination events) were withdrawn and assayed for TreA [phospho-α-(1,1)-glucosidase] reporter enzyme activity using the chromogenic substrate α-PNPG (Gotsche and Dahl, 1995). TreA enzyme activity is expressed in units per milligram of protein. Protein concentrations of cell extracts were estimated from the optical density of the cultures (Miller, 1972).

### Buffer-Screen to Improve the Stability of the GbsR Protein

The previously used recombinant GbsR protein contained a *Strep*-tag II affinity peptide at its N-terminus to allow its purification by affinity chromatography. The buffer used for the purification of the *Strep*-tag II-GbsR protein contained 100 mM Tris−HCl (pH 7.5) and 150 mM NaCl (Nau-Wagner et al., 2012). In our hands, this particular recombinant protein when re-suspended in the indicated solution was difficult to handle experimentally because it tended to aggregate. We therefore constructed a new expression plasmid (pSTH02) that yielded a recombinant GbsR protein with a *Strep*-tag II affinity peptide fused to the C-terminus of GbsR (Supplementary Table S5). To improve the solubility and stability of the GbsR-*Strep*-tag II protein, we carried out an extensive buffer screen (192 conditions) using a nano-differential scanning fluorimetry procedure that measures changes in the intrinsic Trp-fluorescens, at 330 nm and 350 nm, during thermal unfolding of the protein. The resulting T_m_ value is defined by the inflection point of the thermal unfolding curve calculated by the ratio of F_350_/F_330_. For these experiments, we used the Prometheus NT.48 analyzer (NanoTemper Technologies GmbH, München, Germany) and the Solubility & Stability Screen and the Solubility & Stability Screen 2 from Hampton Research (Aisa Viejo, CA, United States) in a high-throughput fashion using 96 well plates. For these screens, 5 μl of the GbsR-*Strep*-tag II protein [concentration of 40 – 45 μM in 100 mM Tris−HCl (pH 7.5), 150 mM NaCl] was mixed with 20 μl of the various buffer solutions and the denaturation of the protein was followed in a temperature range between 20°C and 95°C by applying a linear temperature increase of 2°C min^–1^. The resulting dataset is documented in Supplementary Figure S6. While we were not able to determine a reliable T_m_ for the GbsR-*Strep*-tag II resuspended in the buffer mentioned above (Nau-Wagner et al., 2012), a number of buffer conditions lead to a significant improvement in the stability of the recombinant protein (Supplementary Figure S6). Based upon these results, we chose to change the buffer conditions for the purification procedure of the recombinant GbsR-*Strep*-tag II protein to a buffer containing 100 mM potassium phosphate (pH 8) and 300 mM NaCl.

### Overproduction and Purification of Recombinant GbsR Protein From *Bacillus subtilis*

Overproduction of the GbsR-*Strep*-tag II protein was carried out in the *E. coli B* strain BL21 harboring plasmid pSTH02, a derivative of the expression vector pASG-IBA3 (IBA, Göttingen, Germany). For this purpose, *E. coli* BL21 harboring plasmid pSTH02 was grown at 37°C in MMA (Miller, 1972) supplemented with 0.5% glucose (wt/vol) as a carbon source, 0.5% casamino acids (wt/vol), 1 mg l^–1^ thiamine, and 1 mM MgSO_4_. The medium contained the antibiotic ampicillin (100 μg ml^–1^) to select for the presence of plasmid pSTH02. Main cultures were inoculated (to an OD_578_ of 0.1) from pre-cultures grown over night in LB medium. Enhanced transcriptional activity of the *tet* promoter positioned in front of the *gbsR* gene was induced by adding the synthetic inducer AHT of the TetR repressor to the cells to a final concentration of 0.2 μg ml^–1^ when the culture had reached an OD_578_ of 0.5. After additional 2 h of growth, cells were harvested by centrifugation (4800 × *g*, 20 min, 4°C). Cells were re-suspended in a lysis buffer [100 mM potassium phosphate (pH 8), 300 mM NaCl, 2 mM dithiothreitol, 0.4 mM EDTA, 0.5 mM Pefabloc SC, 0.5 mM benzamidine] and disrupted by passaging them several times through a French pressure cell (French^®^ Pressure Cell Press; American Instrument Company, Silver Spring, MD, United States) at 1 000 psi. Centrifugation (18000 rpm, 40 min, 4°C; Hettich Mikro 22R centrifuge equipped with a 18000 rpm 24 × 3g rotor) yielded a cleared cell lysate, which was loaded onto a streptactin column for affinity purification of the GbsR-*Strep*-tag II by using a buffer containing 100 mM potassium phosphate (pH 8) and 300 mM NaCl. A solution of 100 mM potassium phosphate (pH 8), 300 mM NaCl, and 2.5 mM desthiobiotin was used for the elution of the GbsR-*Strep* tag II protein from the streptactin affinity column. The purity of GbsR-*Strep*-tag II protein preparations were assessed by SDS-polyacrylamide gel electrophoresis (15%) (SDS−PAGE); proteins were stained with InstantBlueTM (Sigma-Aldrich, Steinheim, Germany). The freshly purified GbsR-*Strep*-tag II protein was used immediately for all subsequent biochemical experiments.

### Determination of the Quaternary Assembly of the Purified GbsR Protein

The quaternary assembly of the affinity purified GbsR-*Strep* tag II protein was assessed by size-exclusion chromatography. Immediately after purification, 2-ml protein solution (1 mg ml^–1^) was loaded onto a size-exclusion chromatography column (HiLoad 16/600 Superdex 200 pg; GE Healthcare, München, Germany) that was run in a buffer containing 100 mM potassium phosphate (pH 8) and 300 mM NaCl. Thyroglobulin (667 kDa), albumin (66 kDa), ovalbumin (43 kDa), and cytochrome C (12.4 kDa) were used to standardize the size-exclusion chromatography column.

### Electrophoretic Mobility Shift Assays

Electrophoretic Mobility Shift Assays (EMSAs) were carried out to verify the genetically defined DNA-binding site of the GbsR repressor protein. A 96 bp DNA fragment of the *gbsAB* regulatory region was amplified via PCR from genomic DNA of *B. subtilis* JH642 using the primers BS_gbsA_for and BS_gbsA_rev_Dy781, which was labeled with the Dyomics 781 fluorescent dye (Microsynth AG, Balgach, Switzerland) at the 5′-end (Supplementary Table S4). A fragment with a deletion of the putative GbsR operator sequence was generated from plasmid pDH2 5.2 (Supplementary Table S5). Using the above–mentioned set of primers. DNA binding assays were performed by incubating 1 pmol of the desired DNA fragments with various concentrations of the purified GbsR–*Strep* tag II protein in a buffer containing 10 mM Tris (pH 8), 150 μg ml^–1^ herring sperm DNA, and 7.5% (vol/vol) glycerol, in a total reaction volume of 20 μl. After incubation of the reaction mixture for 20 min at 25°C, samples were electrophoretically separated in a native 8% polyacrylamide gel run at 110 V for 45 min. The fluorescent label was detected using an Odyssey FC Imaging System (LI-COR Biosciences, Linoln, United States).

### Data-Base Searches and Bioinformatics

Proteins homologous to the GbsA protein of *B. subtilis* (Boch et al., 1996) were searched within members of the genus *Bacillus* via the Web server of the Department of Energy Joint Genome Institute (JGI^1^) using the BLAST-P algorithm provided through bioinformatics resources of the IMG/MER database (Chen et al., 2019). We restricted our analysis to only one representative from each species/strain, and also analyzed only those genome sequences of which 16S DNA sequences, deposited in the SILVA database (Glöckner et al., 2017), were provided through the IMG/MER web-site. This targeted search resulted in a final data set of 173 Bacilli (Teichmann et al., 2018). Using the gene neighborhood tool^2^ provided by the IMG/MER database (Chen et al., 2019), the genome context in the proximity of *gbsA*-type genes was manually evaluated. Only orthologs that also showed a *gbsB*-like gene encoded in the same gene cluster (*gbsAB*) (Boch et al., 1996) were selected for an alignment of their promoter region using the MAFFT Web-server^3^ (Katoh et al., 2017). The phylogenomic distribution of OpuB- and OpuC-type substrate-binding-protein-dependent ABC transporters from *B. subtilis* (Hoffmann and Bremer, 2017) was evaluated using the amino acid sequence of the respective substrate-binding proteins (OpuBC, OpuCC) (Du et al., 2011; Pittelkow et al., 2011) as the query for a BLAST search (Altschul et al., 1990). In the resulting dataset, a particular ABC transporter was assigned to OpuB- or OpuC-type transporters by comparing the degree of amino-acid conservation of the substrate binding protein relative to that of the *B. subtilis* OpuBC or OpuCC proteins (Kappes et al., 1999) and diagnostic differences in the amino acid residues forming the substrate-binding pockets in these proteins (Du et al., 2011; Pittelkow et al., 2011).

GbsR-related proteins were searched by using the amino acid sequence of the *B. subtilis* GbsR protein as the query sequence (Nau-Wagner et al., 2012) in the IMG/MER database (Chen et al., 2019). The immediate vicinity of *gbsR*-related genes was then manually evaluated to allow their assignment to a specific sub-group of the GbsR-, OpuAR-, YvaV- or, OpcR-type within the GbsR regulatory protein family (Ronzheimer et al., 2018). A phylogenetic tree was constructed based on a 16S rDNA alignment of the chosen *Bacillus* species/strains using the Distance Tree Tool^4^ provided by the IMG/MER Web server (Chen et al., 2019). Two genomes of the genus *Clostridium* were used as an out-group for the construction of the tree as described previously (Teichmann et al., 2018). The amino acid sequences of the GbsR, OpcR and YvaV proteins, as well as the nucleotide sequences of the *gbsAB, opuB*, and *opuC* promoter regions were aligned using the MAFFT Web server with standard bootstrap settings (100 bootstraps) automatically chosen by the Web server^3^ (Katoh et al., 2017). Data concerning the identity of the identified proteins in the various *Bacillus* species are summarized in Supplementary Data Sheet 1.

## Data Availability Statement

All datasets presented in this study are included in the article/Supplementary Material.

## Author Contributions

EB designed and supervised the study. BW, SR, S-AF, AS, and TH conducted the experiments, collected the data, and interpreted the results. BW, TH, and EB wrote the manuscript with input from the other authors. All authors contributed to the article and approved the submitted version.

## Conflict of Interest

The authors declare that the research was conducted in the absence of any commercial or financial relationships that could be construed as a potential conflict of interest.

## Footnotes

1 http://genome.jgi.doe.gov

2 http://img.jgi.doegov/cgi-bin/pub/main.cgi/

3 http://mafft.cbrc.jp/alignment/server/

4 https://img.jgi.doe.gov/cgi-bin/m/main.cgi?section=DistanceTree&page=tree

## References

[B1] Alekshun M. N. Levy S. B. (1997). Regulation of chromosomally mediated multiple antibiotic resistance: the mar regulon. *Antimicrob. Agents Chemother.* 41 2067–2075. 10.1128/aac.41.10.2067 9333027 PMC164072

[B2] Altschul S. F. Gish W. Miller W. Myers E. W. Lipman D. J. (1990). Basic local alignment search tool. *J. Mol. Biol.* 215 403–410. 2231712 10.1016/S0022-2836(05)80360-2

[B3] Barth S. Huhn M. Matthey B. Klimka A. Galinski E. A. Engert A. (2000). Compatible-solute-supported periplasmic expression of functional recombinant proteins under stress conditions. *Appl. Environ. Microbiol.* 66 1572–1579. 10.1128/aem.66.4.1572-1579.2000 10742244 PMC92025

[B4] Bervoets I. Charlier D. (2019). Diversity, versatility and complexity of bacterial gene regulation mechanisms: opportunities and drawbacks for applications in synthetic biology. *FEMS Microbiol. Rev.* 43 304–339. 10.1093/femsre/fuz001 30721976 PMC6524683

[B5] Boch J. Kempf B. Bremer E. (1994). Osmoregulation in *Bacillus subtilis*: synthesis of the osmoprotectant glycine betaine from exogenously provided choline. *J. Bacteriol.* 176 5364–5371. 10.1128/jb.176.17.5364-5371.1994 8071213 PMC196722

[B6] Boch J. Kempf B. Schmid R. Bremer E. (1996). Synthesis of the osmoprotectant glycine betaine in *Bacillus subtilis*: characterization of the gbsAB genes. *J. Bacteriol.* 178 5121–5129. 10.1128/jb.178.17.5121-5129.1996 8752328 PMC178307

[B7] Boch J. Nau-Wagner G. Kneip S. Bremer E. (1997). Glycine betaine aldehyde dehydrogenase from *Bacillus subtilis*: characterization of an enzyme required for the synthesis of the osmoprotectant glycine betaine. *Arch. Microbiol.* 168 282–289. 10.1007/s002030050500 9297465

[B8] Bolen D. W. Baskakov I. V. (2001). The osmophobic effect: natural selection of a thermodynamic force in protein folding. *J. Mol. Biol.* 310 955–963. 10.1006/jmbi.2001.4819 11502004

[B9] Bourot S. Sire O. Trautwetter A. Touze T. Wu L. F. Blanco C. (2000). Glycine betaine-assisted protein folding in a *lysA* mutant of *Escherichia coli*. *J. Biol. Chem.* 275 1050–1056. 10.1074/jbc.275.2.1050 10625645

[B10] Branda S. S. Gonzalez-Pastor J. E. Ben-Yehuda S. Losick R. Kolter R. (2001). Fruiting body formation by *Bacillus subtilis*. *Proc. Natl. Acad. Sci. U.S.A.* 98 11621–11626. 10.1073/pnas.191384198 11572999 PMC58779

[B11] Bremer E. Krämer R. (2019). Responses of microorganisms to osmotic stress. *Annu. Rev. Microbiol.* 73 313–314. 31180805 10.1146/annurev-micro-020518-115504

[B12] Brill J. Hoffmann T. Bleisteiner M. Bremer E. (2011). Osmotically controlled synthesis of the compatible solute proline is critical for cellular defense of *Bacillus subtilis* against high osmolarity. *J. Bacteriol.* 193 5335–5346. 10.1128/JB.05490-11 21784929 PMC3187420

[B13] Browne H. P. Anvar S. Y. Frank J. Lawley T. D. Roberts A. P. Smits W. K. (2015). Complete genome sequence of BS49 and draft genome sequence of BS34A, *Bacillus subtilis* strains carrying Tn916. *FEMS Microbiol. Lett.* 362 1–4. 10.1093/femsle/fnu050 25673660

[B14] Chen I. A. Chu K. Palaniappan K. Pillay M. Ratner A. Huang J. (2019). IMG/M v.5.0: an integrated data management and comparative analysis system for microbial genomes and microbiomes. *Nucleic Acids Res.* 47 D666–D677. 10.1093/nar/gky901 30289528 PMC6323987

[B15] da Costa M. S. Santos H. Galinski E. A. (1998). An overview of the role and diversity of compatible solutes in Bacteria and Archaea. *Adv. Biochem. Eng. Biotechnol.* 61 117–153. 10.1007/bfb0102291 9670799

[B16] Dai X. Zhu M. (2018). High osmolarity modulates bacterial cell size through reducing initiation volume in *Escherichia coli*. *mSphere* 3:e00430-18. 10.1128/mSphere.00430-18 30355666 PMC6200984

[B17] de Lima Alves F. Stevenson A. Baxter E. Gillion J. L. Hejazi F. Hayes S. (2015). Concomitant osmotic and chaotropicity-induced stresses in *Aspergillus wentii*: compatible solutes determine the biotic window. *Curr. Genet.* 61 457–477. 10.1007/s00294-015-0496-8 26055444

[B18] Dinnbier U. Limpinsel E. Schmid R. Bakker E. P. (1988). Transient accumulation of potassium glutamate and its replacement by trehalose during adaptation of growing cells of *Escherichia coli* K-12 to elevated sodium chloride concentrations. *Arch. Microbiol.* 150 348–357. 10.1007/bf00408306 3060036

[B19] Du Y. Shi W. W. He Y. X. Yang Y. H. Zhou C. Z. Chen Y. (2011). Structures of the substrate-binding protein provide insights into the multiple compatible solute binding specificities of the *Bacillus subtilis* ABC transporter OpuC. *Biochem. J.* 436 283–289. 10.1042/BJ20102097 21366542

[B20] Dubendorff J. W. Studier F. W. (1991). Controlling basal expression in an inducible T7 expression system by blocking the target T7 promoter with lac repressor. *J. Mol. Biol.* 219 45–59. 10.1016/0022-2836(91)90856-2 1902522

[B21] Evans K. Adewoye L. Poole K. (2001). MexR repressor of the *mexAB-oprM* multidrug efflux operon of *Pseudomonas aeruginosa*: identification of MexR binding sites in the *mexA-mexR* intergenic region. *J. Bacteriol.* 183 807–812. 10.1128/JB.183.3.807-812.2001 11208776 PMC94945

[B22] Galan B. Kolb A. Sanz J. M. Garcia J. L. Prieto M. A. (2003). Molecular determinants of the hpa regulatory system of *Escherichia coli*: the HpaR repressor. *Nucleic Acids Res.* 31 6598–6609. 10.1093/nar/gkg851 14602920 PMC275547

[B23] Glöckner F. O. Yilmaz P. Quast C. Gerken J. Beccati A. Ciuprina A. (2017). 25 years of serving the community with ribosomal RNA gene reference databases and tools. *J. Biotechnol.* 261 169–176. 10.1016/j.jbiotec.2017.06.1198 28648396

[B24] Gonzalez-Pastor J. E. Hobbs E. C. Losick R. (2003). Cannibalism by sporulating bacteria. *Science* 301 510–513. 10.1126/science.1086462 12817086

[B25] Gotsche S. Dahl M. K. (1995). Purification and characterization of the phospho-alpha-(1,1)-glucosidase (TreA) of *Bacillus subtilis* 168. *J. Bacteriol.* 177 2721–2726. 10.1128/jb.177.10.2721-2726.1995 7751281 PMC176942

[B26] Grove A. (2013). MarR family transcription factors. *Curr. Biol.* 23 R142–R143. 10.1016/j.cub.2013.01.013 23428319

[B27] Grove A. (2017). Regulation of metabolic pathways by MarR family transcription factors. *Comput. Struct. Biotechnol. J.* 15 366–371. 10.1016/j.csbj.2017.06.001 28694934 PMC5487221

[B28] Guerout-Fleury A. M. Frandsen N. Stragier P. (1996). Plasmids for ectopic integration in *Bacillus subtilis*. *Gene* 180 57–61. 10.1016/s0378-1119(96)00404-0 8973347

[B29] Gunde-Cimerman N. Plemenitas A. Oren A. (2018). Strategies of adaptation of microorganisms of the three domains of life to high salt concentrations. *FEMS Microbiol. Rev.* 42 353–375. 10.1093/femsre/fuy009 29529204

[B30] Gupta A. Pande A. Sabrin A. Thapa S. S. Gioe B. W. Grove A. (2019). MarR family transcription factors from Burkholderia species: hidden clues to control of virulence-associated genes. *Microbiol. Mol. Biol. Rev.* 83:e00039-18. 10.1128/MMBR.00039-18 30487164 PMC6383443

[B31] Hahne H. Mäder U. Otto A. Bonn F. Steil L. Bremer E. (2010). A comprehensive proteomics and transcriptomics analysis of *Bacillus subtilis* salt stress adaptation. *J. Bacteriol.* 192 870–882. 10.1128/JB.01106-09 19948795 PMC2812467

[B32] Harwood C. R. Cutting S. M. (1990). *Molecular Biological Methods for Bacillus.* Chichester: John Wiley & Sons.

[B33] Hoffmann T. Bleisteiner M. Sappa P. K. Steil L. Mäder U. Völker U. (2017). Synthesis of the compatible solute proline by *Bacillus subtilis*: point mutations rendering the osmotically controlled *proHJ* promoter hyperactive. *Environ. Microbiol.* 19 3700–3720. 10.1111/1462-2920.13870 28752945

[B34] Hoffmann T. Bremer E. (2011). Protection of *Bacillus subtilis* against cold stress via compatible-solute acquisition. *J. Bacteriol.* 193 1552–1562. 10.1128/JB.01319-10 21296969 PMC3067655

[B35] Hoffmann T. Bremer E. (2016). “Management of osmotic stress by *Bacillus subtilis*: genetics and physiology,” in *Stress and Environmental Regulation of Gene Expression and Adaptation in Bacteria*, ed. de Bruijn F. J. (Hoboken, NJ: Wiley-Blackwell Publishers), 657–676. 10.1002/9781119004813.ch63

[B36] Hoffmann T. Bremer E. (2017). Guardiens in a stressful world: the Opu family of compatible solute transporters from *Bacillus subtilis*. *Biol. Chem.* 398 193–214. 10.1515/hsz-2016-0265 27935846

[B37] Hoffmann T. Warmbold B. Smits S. H. J. Tschapek B. Ronzheimer S. Bashir A. (2018). Arsenobetaine: an ecophysiologically important organoarsenical confers cytoprotection against osmotic stress and growth temperature extremes. *Environ. Microbiol.* 20 305–323. 10.1111/1462-2920.13999 29159878

[B38] Hoffmann T. Wensing A. Brosius M. Steil L. Völker U. Bremer E. (2013). Osmotic control of *opuA* expression in *Bacillus subtilis* and its modulation in response to intracellular glycine betaine and proline pools. *J. Bacteriol.* 195 510–522. 10.1128/JB.01505-12 23175650 PMC3554007

[B39] Ignatova Z. Gierasch L. M. (2006). Inhibition of protein aggregation in vitro and in vivo by a natural osmoprotectant. *Proc. Natl. Acad. Sci. U.S.A.* 103 13357–13361. 10.1073/pnas.0603772103 16899544 PMC1569168

[B40] Kappes R. M. Kempf B. Kneip S. Boch J. Gade J. Meier-Wagner J. (1999). Two evolutionarily closely related ABC transporters mediate the uptake of choline for synthesis of the osmoprotectant glycine betaine in *Bacillus subtilis*. *Mol. Microbiol.* 32 203–216. 10.1046/j.1365-2958.1999.01354.x 10216873

[B41] Katoh K. Rozewicki J. Yamada K. D. (2017). MAFFT online service: multiple sequence alignment, interactive sequence choice and visualization. *Brief. Bioinform.* 20 1160–1166. 10.1093/bib/bbx108 28968734 PMC6781576

[B42] Kempf B. Bremer E. (1998). Uptake and synthesis of compatible solutes as microbial stress responses to high osmolality environments. *Arch. Microbiol.* 170 319–330. 10.1007/s002030050649 9818351

[B43] Kunst F. Ogasawara N. Moszer I. Albertini A. M. Alloni G. Azevedo V. (1997). The complete genome sequence of the gram-positive bacterium *Bacillus subtilis*. *Nature* 390 249–256. 10.1038/36786 9384377

[B44] Kuwayama H. Obara S. Morio T. Katoh M. Urushihara H. Tanaka Y. (2002). PCR-mediated generation of a gene disruption construct without the use of DNA ligase and plasmid vectors. *Nucleic Acids Res.* 30:E2. 10.1093/nar/30.2.e2 PMC99841 11788728

[B45] Lee C. H. Wu T. Y. Shaw G. C. (2013). Involvement of OpcR, a GbsR-type transcriptional regulator, in negative regulation of two evolutionarily closely related choline uptake genes in *Bacillus subtilis*. *Microbiology* 159 2087–2096. 10.1099/mic.0.067074-0 23960087

[B46] Leyn S. A. Kazanov M. D. Sernova N. V. Ermakova E. O. Novichkov P. S. Rodionov D. A. (2013). Genomic reconstruction of the transcriptional regulatory network in *Bacillus subtilis*. *J. Bacteriol.* 195 2463–2473. 10.1128/JB.00140-13 23504016 PMC3676070

[B47] Mandic-Mulec I. Stefanic P. van Elsas J. D. (2015). Ecology of Bacillaceae. *Microbiol. Spectr.* 3:TBS-0017-2013. 10.1128/microbiolspec.TBS-0017-2013 26104706

[B48] Miller J. H. (1972). *Experiments in Molecular Genetics.* Cold Spring Harbor, NY: Cold Spring Harbor Laboratory.

[B49] Nau-Wagner G. Opper D. Rolbetzki A. Boch J. Kempf B. Hoffmann T. (2012). Genetic control of osmoadaptive glycine betaine synthesis in *Bacillus subtilis* through the choline-sensing and glycine betaine-responsive GbsR repressor. *J. Bacteriol.* 194 2703–2714. 10.1128/jb.06642-11 22408163 PMC3347207

[B50] Nicolas P. Mäder U. Dervyn E. Rochat T. Leduc A. Pigeonneau N. (2012). Condition-dependent transcriptome reveals high-level regulatory architecture in *Bacillus subtilis*. *Science* 335 1103–1106. 10.1126/science.1206848 22383849

[B51] Nye T. M. Schroeder J. W. Kearns D. B. Simmons L. A. (2017). Complete genome sequence of undomesticated *Bacillus subtilis* strain NCIB 3610. *Genome Announc.* 5:e00364-17. 10.1128/genomeA.00364-17 28522717 PMC5477328

[B52] Oren A. (1999). Bioenergetic aspects of halophilism. *Microbiol. Mol. Biol. Rev.* 63 334–348. 10.1128/mmbr.63.2.334-348.1999 10357854 PMC98969

[B53] Pittelkow M. Tschapek B. Smits S. H. Schmitt L. Bremer E. (2011). The crystal structure of the substrate-binding protein OpuBC from *Bacillus subtilis* in complex with choline. *J. Mol. Biol.* 411 53–67. 10.1016/j.jmb.2011.05.037 21658392

[B54] Rath H. Reder A. Hoffmann T. Hammer E. Seubert A. Bremer E. (2020). Management of osmoprotectant uptake hierarchy in *Bacillus subtilis* via a SigB-dependnet antisense RNA. *Font. Microbiol.* 11:622. 10.3389/fmicb.2020.00622 32373088 PMC7186363

[B55] Ray S. S. Bonanno J. B. Chen H. de Lencastre H. Wu S. Tomasz A. (2003). X-ray structure of an *M. jannaschii* DNA-binding protein: implications for antibiotic resistance in *S. aureus*. *Proteins* 50 170–173. 10.1002/prot.10272 12471609

[B56] Record M. T. Jr. Courtenay E. S. Cayley S. Guttman H. J. (1998). Biophysical compensation mechanisms buffering *E. coli* protein-nucleic acid interactions against changing environments. *Trends Biochem. Sci.* 23 190–194. 10.1016/s0968-0004(98)01207-9 9612084

[B57] Reyes-Lamothe R. Sherratt D. J. (2019). The bacterial cell cycle, chromosome inheritance and cell growth. *Nat. Rev. Microbiol.* 17 467–478. 10.1038/s41579-019-0212-7 31164753

[B58] Roeßler M. Müller V. (2001). Osmoadaptation in bacteria and archaea: common principles and differences. *Environ. Microbiol. Rep.* 3 743–754. 10.1046/j.1462-2920.2001.00252.x 11846768

[B59] Ronzheimer S. Warmbold B. Arnhold C. Bremer E. (2018). The GbsR family of transcriptional regulators: functional characterization of the OpuAR repressor. *Front. Microbiol.* 9:2536. 10.3389/fmicb.2018.02536 30405586 PMC6207618

[B60] Schöck F. Gotsche S. Dahl M. K. (1996). Vectors using the phospho-alpha-(1,1)-glucosidase-encoding gene *treA* of *Bacillus subtilis* as a reporter. *Gene* 170 77–80. 10.1016/0378-1119(95)00860-8 8621093

[B61] Smith J. L. Goldberg J. M. Grossman A. D. (2014). Complete genome sequences of *Bacillus subtilis* subsp. *subtilis* laboratory strains JH642 (AG174) and AG1839. *Genome Announc.* 2:e00663-14. 10.1128/genomeA.00663-14 24994804 PMC4082004

[B62] Stadmiller S. S. Gorensek-Benitez A. H. Guseman A. J. Pielak G. J. (2017). Osmotic shock induced protein destabilization in living cells and its reversal by glycine betaine. *J. Mol. Biol.* 429 1155–1161. 10.1016/j.jmb.2017.03.001 28263768 PMC5985519

[B63] Steil L. Hoffmann T. Budde I. Völker U. Bremer E. (2003). Genome-wide transcriptional profiling analysis of adaptation of *Bacillus subtilis* to high salinity. *J. Bacteriol.* 185 6358–6370. 10.1128/jb.185.21.6358-6370.2003 14563871 PMC219388

[B64] Stevenson A. Cray J. A. Williams J. P. Santos R. Sahay R. Neuenkirchen N. (2015). Is there a common water-activity limit for the three domains of life? *ISME J.* 9 1333–1351. 10.1038/ismej.2014.219 25500507 PMC4438321

[B65] Teichmann L. Chen C. Hoffmann T. Smits S. H. J. Schmitt L. Bremer E. (2017). From substrate specificity to promiscuity: hybrid ABC transporters for osmoprotectants. *Mol. Microbiol.* 104 761–780. 10.1111/mmi.13660 28256787

[B66] Teichmann L. Kümmel H. Warmbold B. Bremer E. (2018). OpuF: a new *Bacillus* compatible solute ABC transporter with a substrate-binding protein fused to the trans-membrane domain. *Appl. Environ. Microbiol.* 84:e01728-18. 10.1128/AEM.01728-18 30097444 PMC6182910

[B67] van den Berg J. Boersma A. J. Poolman B. (2017). Microorganisms maintain crowding homeostasis. *Nat. Rev. Microbiol.* 15 309–318. 10.1038/nrmicro.2017.17 28344349

[B68] Whatmore A. M. Chudek J. A. Reed R. H. (1990). The effects of osmotic upshock on the intracellular solute pools of *Bacillus subtilis*. *J. Gen. Microbiol.* 136 2527–2535. 10.1099/00221287-136-12-2527 2127802

[B69] Wood J. M. (2011). Bacterial osmoregulation: a paradigm for the study of cellular homeostasis. *Annu. Rev. Microbiol.* 65 215–238. 10.1146/annurev-micro-090110-102815 21663439

[B70] Yan X. Yu H. J. Hong Q. Li S. P. (2008). Cre/lox system and PCR-based genome engineering in *Bacillus subtilis*. *Appl. Environ. Microbiol.* 74 5556–5562. 10.1128/aem.01156-08 18641148 PMC2546623

[B71] Zeigler D. R. (2011). The genome sequence of *Bacillus subtilis* subsp. *spizizenii* W23: insights into speciation within the *B. subtilis* complex and into the history of *B. subtilis* genetics. *Microbiology* 157 2033–2041. 10.1099/mic.0.048520-0 21527469

