# Supplementary material for: Two MarR-Type Repressors Balance Precursor Uptake and Glycine Betaine Synthesis in Bacillus subtilis to Provide Cytoprotection Against Sustained Osmotic Stress
Source: Front Microbiol. 2020 Jul 23;11:1700. doi: 10.3389/fmicb.2020.01700 (PMC7396694; doi:10.3389/fmicb.2020.01700)
Supplement: Supplementary file 1 [file Data_Sheet_1.PDF]

Supplementary material to

Two MarR-type repressors balance precursor uptake and glycine betaine synthesis in *Bacillus subtilis* to provide cytoprotection against sustained osmotic stress

Bianca Warmbold[1], Stefanie Ronzheimer[1], Sven-Andreas Freibert[2], Andreas Seubert[3], Tamara Hoffmann[1], and Erhard Bremer[1,4,*]

[1]Laboratory for Microbiology, Department of Biology, Philipps-University Marburg, Marburg, Karl-von-Frisch Str. 8, Marburg, D-35043, Germany

[2]Department of Medicine, Institute for Cytobiology and Cytopathology, Philipps-University Marburg, Marburg, Robert-Koch Str. 6, Marburg, D-35032, Germany

[3]Faculty of Chemistry, Analytical Chemistry, Philipps-University Marburg, Marburg, Hans-Meerwein Str. 4, Marburg, D-35043, Germany

[4]Center for Synthetic Microbiology (SYNMIKRO), Philipps-University Marburg, Hans-Meerwein Str. 6, Marburg, D-35043, Germany

Running head: Adaptation to sustained osmotic stress

***Correspondence to Erhard Bremer:** Laboratory for Microbiology, Department of Biology, Philipps-University Marburg, Karl-von-Frisch Strasse 8, D-35043 Marburg, Germany. Phone: (+49)-6421-2821529. Fax: (+49)-6421-2828979. E-Mail: bremer@staff.uni-marburg.de

Table S1. Mutational study of the previously predicted GbsR binding site in the *gbsAB* regulatory region.

Promoter region Φ(*gbsA-treA*)	Strain	*gbsR*	TreA activity [U (mg protein)$^{-1}$] Non-induced	Induced (NaCl + Cho)
TTTTATTTAACAAACTTTATTTA	DHB4	+	4 ± 1	110 ± 2
	DHB12	-	137 ± 4	153 ± 15
TTTTATTTAACAAAGTTTATTTA	AROB9	+	4 ± 2	99 ± 3
	AROB4	-	113 ± 15	151 ± 5
TTTTATTTAACAAACTTCATTTA	TMB128	+	6 ± 0	122 ± 8
	TMB131	-	126 ± 23	171 ± 21
TTTTATTTAACAAACTTTAATTA	TMB129	+	4 ± 1	112 ± 22
	TMB132	-	149 ± 11	181 ± 12
TTTTATTTAACAAACTTTATATA	TMB130	+	9 ± 1	161 ± 21
	TMB133	-	103 ± 5	129 ± 12
TTTTATTTAACAAAGTTCAAATA	AROB10	+	7 ± 2	105 ± 4
	AROB5	-	145 ± 27	161 ± 14

The GbsR binding site, previously suggested by Nau-Wagner et al. (2012) for the *gbsAB* operon is underlined (Nau-Wagner et al., 2012). Substitutions within this sequence that were generated through site-directed mutagenesis are marked in red. *B. subtilis* strains carrying *gbsA-treA* operon fusions with the indicated mutations of the putative GbsR binding site were grown in minimal medium (SMM) without NaCl to early log phase (OD_{578} of 0.25; non-induced). After the addition of 0.4 M NaCl and 1 mM choline (final concentrations) to the cultures, the cells were further grown for 90 min. (induced). Samples from both time points were assayed for their TreA reporter enzyme activity.

Table S2. *B. subtilis* strains used in this study.

Strain	Relevant genotype	Reference/source
JH642	*trpC2 pheA1*	J. Hoch; BGSC[a)] 1A96
AROB4	*Δ(treA::erm)2 (gbsR::neo)1 [amyE::Φ(gbsA´-treA)8]*	This study
AROB5	*Δ(treA::erm)2 (gbsR::neo)1 [amyE::Φ(gbsA´-treA)9]*	This study
AROB9	*Δ(treA::erm)2 [amyE::Φ(gbsA´-treA)8]*	This study
AROB10	*Δ(treA::erm)2 [amyE::Φ(gbsA´-treA)9]*	This study
BWB23	*Δ(treA::erm)1 (gbsR::neo)1 amyE::[Φ(opuB´-treA)1]*	This study
BWB25	*Δ(treA::erm)2 [amyE::Φ(opuBA´-treA)2]*	This study
BWB26	*Δ(treA::erm)2 [amyE::Φ(opuBA´-treA)3]*	This study
BWB27	*Δ(treA::erm)2 [amyE::Φ(opuBA´-treA)4]*	This study
BWB28	*Δ(treA::erm)2 [amyE::Φ(opuBA´-treA)5]*	This study
BWB29	*Δ(treA::erm)2 (gbsR::neo)1 [amyE::Φ(opuBA´-treA)2]*	This study
BWB30	*Δ(treA::erm)2 (gbsR::neo)1 [amyE::Φ(opuBA´-treA)3]*	This study
BWB31	*Δ(treA::erm)2 (gbsR::neo)1 [amyE::Φ(opuBA´-treA)4]*	This study
BWB32	*Δ(treA::erm)2 (gbsR::neo)1 [amyE::Φ(opuBA´-treA)5]*	This study
BWB33	*Δ(treA::erm)2 [amyE::Φ(opuBA´-treA)6]*	This study
BWB34	*Δ(treA::erm)2 [amyE::Φ(opuBA´-treA)7]*	This study
BWB35	*Δ(treA::erm)2 (gbsR::neo)1 [amyE::Φ(opuBA´-treA)6]*	This study
BWB36	*Δ(treA::erm)2 (gbsR::neo)1 [amyE::Φ(opuBA´-treA)5]*	This study
BWB127	*Δ(treA::erm)2 [amyE::Φ(opcR´-treA)1]1*	This study
BWB130	*Δ(treA::erm)2 [amyE::Φ(opcR´-treA)1]1* Δ*(gbsR::spc)2*	This study
BWB131	*Δ(treA::erm)2 [amyE::*Φ*(opcR´-treA)1]1* Δ(*opcR::zeo*)2	This study
BWB132	*Δ(treA::erm)2 [amyE::*Φ*(opcR´-treA)1]1* Δ(*yvaV::tet*)2	This study
DHB2	*Δ(treA::erm)2 [amyE::Φ(gbsR´-treA)1]*	This study
TMB647	*Δ(treA::erm)2 [amyE::Φ(gbsR´-treA)1]* Δ*(gbsR::spc)2*	This study
TMB648	*Δ(treA::erm)2 [amyE::Φ(gbsR´-treA)1]* Δ(*yvaV::tet*)2	This study
TMB649	*Δ(treA::erm)2 [amyE::Φ(gbsR´-treA)1]* Δ(*opcR::zeo*)2	This study
DHB4	*Δ(treA::erm)2 [amyE::Φ(gbsA´-treA)1*	(Nau-Wagner *et al.*, 2012)
DHB12	*Δ(treA::erm)2 (gbsR::neo)1 [amyE::Φ(gbsA´-treA)1]*	(Nau-Wagner *et al.*, 2012)
GNB37	*Δ(treA::erm)2*	(Nau-Wagner *et al.*, 2012)
STHB01	*Δ(opcR::zeo)1*	This study
STHB07	*Δ(treA::erm)2 Δ(opcR::zeo)2* Δ*(yvaV::tet)3*	(Ronzheimer *et al.*, 2018)
STHB08	*Δ(treA::erm)2 Δ(opcR::zeo)2*	(Ronzheimer *et al.*, 2018)
STHB09	*Δ(treA::erm)2 Δ(yvaV::tet)2*	This study
STHB14	*Δ(gbsR::spc)1*	This study
STHB15	*Δ(treA::erm)2 Δ(gbsR::spc)2*	This study
STHB16	*Δ(treA::erm)2 Δ(opcR::zeo)2 Δ(gbsR::spc)3*	This study
STHB17	*Δ(treA::erm)2 Δ(yvaV::tet)2 Δ(gbsR::spc)3*	This study
STHB18	*Δ(treA::erm)2 Δ(opcR::zeo)2 Δ(yvaV::tet)3 Δ(gbsR::spc)4*	This study
STHB33	*Δ(treA::erm)2 [amyE::ΦopuCA´-treA]1*	This study

[a)] BGSC: Bacillus Genetic Stock Center (Columbus, OH, USA). The genome sequence of this strain has been reported (Smith et al., 2014).

Table S2. *B. subtilis* strains used in this study (continuation).

Strain	Relevant genotype	Reference/source
STHB34	*Δ(treA::erm)2 Δ(opcR::zeo)2 Δ(yvaV::tet)3 [amyE::ΦopuCA´-treA]1*	This study
STHB35	*Δ(treA::erm)2 Δ(opcR::zeo)2 [amyE::ΦopuCA´-treA]1*	This study
STHB36	*Δ(treA::erm)2 Δ(yvaV::tet)2 [amyE::ΦopuCA´-treA]1*	This study
STHB37	*Δ(treA::erm)2 Δ(gbsR::spc)2 [amyE::ΦopuCA´-treA]1*	This study
STHB38	*Δ(treA::erm)2 Δ(opcR::zeo)2 Δ(gbsR::spc)3 [amyE::ΦopuCA´-treA]1*	This study
STHB39	*Δ(treA::erm)2 Δ(yvaV::tet)2 Δ(gbsR::spc)3 [amyE::ΦopuCA´-treA]1*	This study
STHB40	*Δ(treA::erm)2 Δ(opcR::zeo)2 Δ(yvaV::tet)3 Δ(gbsR::spc)4 [amyE::ΦopuCA´-treA]1*	This study
STHB49	*Δ(treA::erm)2 [amyE::ΦopuBA´-treA]1*	This study
STHB50	*Δ(treA::erm)2 Δ(opcR::zeo)2 Δ(yvaV::tet)3 [amyE::ΦopuBA´-treA]1*	This study
STHB51	*Δ(treA::erm)2 Δ(opcR::zeo)2 [amyE::ΦopuBA´-treA]1*	This study
STHB52	*Δ(treA::erm)2 Δ(yvaV::tet)2 [amyE::ΦopuBA´-treA]1*	This study
STHB53	*Δ(treA::erm)2 Δ(gbsR::spc)2 [amyE::ΦopuBA´-treA]1*	This study
STHB54	*Δ(treA::erm)2 Δ(opcR::zeo)2 Δ(gbsR::spc)3 [amyE::ΦopuBA´-treA]1*	This study
STHB55	*Δ(treA::erm)2 Δ(yvaV::tet)2 Δ(gbsR::spc)3 [amyE::ΦopuBA´-treA]1*	This study
STHB56	*Δ(treA::erm)2 Δ(opcR::zeo)2 Δ(yvaV::tet)3 Δ(gbsR::spc)4 [amyE::ΦopuBA´-treA]1*	This study
STHB78	*Δ(treA::erm)2 [amyE::Φ(gbsA´-treA)2]*	This study
STHB79	*Δ(treA::erm)2 [amyE::Φ(gbsA´-treA)3]*	This study
STHB80	*Δ(treA::erm)2 [amyE::Φ(gbsA´-treA)4]*	This study
STHB82	*Δ(treA::erm)2 [amyE: Φ(gbsA´-treA)5]*	This study
STHB83	*Δ(treA::erm)2 [amyE: Φ(gbsA´-treA)6]*	This study
STHB84	*Δ(treA::erm)2 [amyE::Φ(gbsA´-treA)7]*	This study
STHB85	*Δ(treA::erm)2 (gbsR::neo)1 [amyE::Φ(gbsA´-treA)2]*	This study
STHB86	*Δ(treA::erm)2 (gbsR::neo)1 [amyE::Φ(gbsA´-treA)3]*	This study
STHB87	*Δ(treA::erm)2 (gbsR::neo)1 [amyE::Φ(gbsA´-treA)4]*	This study
STHB89	*Δ(treA::erm)2 (gbsR::neo)1 [amyE::Φ(gbsA´-treA)5]*	This study
STHB90	*Δ(treA::erm)2 (gbsR::neo)1 [amyE::Φ(gbsA´-treA)6]*	This study
STHB91	*Δ(treA::erm)2 (gbsR::neo)1 [amyE::Φ(gbsA´-treA)7]*	This study
TMB118	*Δ(opuA::tet)3 Δ(opuC::spc)3 Δ(opuD::neo)2 Δ(opuB::erm)3*	(Teichmann et al., 2017)
TMB128	*Δ(treA::erm)2 [amyE::Φ(gbsA´-treA)10]*	This study
TMB129	*Δ(treA::erm)2 [amyE::Φ(gbsA´-treA)11]*	This study
TMB130	*Δ(treA::erm)2 [amyE::Φ(gbsA´-treA)12]*	This study
TMB131	*Δ(treA::erm)2 (gbsR::neo)1 [amyE::Φ(gbsA´-treA)10]*	This study
TMB132	*Δ(treA::erm)2 (gbsR::neo)1 [amyE::Φ(gbsA´-treA)11]*	This study
TMB133	*Δ(treA::erm)2 (gbsR::neo)1 [amyE::Φ(gbsA´-treA)12]*	This study

Table S3. Osmotic induction of *opuB*, *opuC*, and *opcR* expression.

Medium	Osmolarity (mosmol kg^{-1})[a]	TreA activity [U (mg protein)$^{-1}$][b]		
		Φ(*opuBA-treA*)	Φ(*opuCA-treA*)	Φ(*opcR-treA*)
SMM	356	33 ± 1	133 ± 5	17 ± 1
SMM 0.68 M glycerol	1100	13 ± 2	95 ± 8	10 ± 1
SMM 0.4 M NaCl	1188	81 ± 2	278 ± 4	21 ± 0
SMM 0.4 M KCl	1178	93 ± 3	247 ± 5	27 ± 4
SMM 0.62 M sucrose	1118	89 ± 20	304 ± 24	35 ± 3

[a] The osmolarities of the different growth media were taken from the literature (Hoffmann et al., 2013)

[b] The *B. subtilis* reporter fusion strains STHB49 (*opuBA-treA*), STHB33 (*opuCA-treA*) and, BWB127 (*opcR-treA*) were grown in the indicated media to mid-exponential growth phase (OD_{578} 1-1.5) and samples were assayed for TreA reporter enzyme activity. The given data are the mean and standard deviations of four independent biological replicates, which were each assayed twice.

Table S4. Oligonucleotides used in this study.

Primer name	Primer sequence (5´-3´)[a)]	Resulting plasmid / application
SmaI-opuB for	AAACCCGGGCAACGGTTTCATCCTTTCAGC	pSTH67
BglII-opuB rev	AAAAGATCTGTTCAACATCCGGGCTGGA	
OpuC TreA1 for	AAACCCGGGCACAGCTGATCATCCCTTCA	pSTH62
OpuC TreA rev	AAAGGATCCCCCGCTCGATATCCGGTC	
OpcR-treA_for	CTACCCGGGGCAAGCTTAATCGCTTCATCC	pBW34
OpcR-treA_rev	GATGGATCCCTGGCTCATCCGTGTTTTGC	
GbsR-treA_for	AAACCCGGGCTGCCAAGCCGGCGTAATAT	pGNB10
GbsR-treA_rev	AAAGGATCCGATATCCTCATCGAGATCTTCC	
gbsR_B.sub_IBA3_for	AAGCTCTTCAATGGATGAAAATCCAGAATTTGCAGCT	pSTH02
gbsR_B.sub_IBA3_rev	AAGCTCTTCACCCCTTTGTTTCGACCGGTATAAATTTA AAA	
opuB_mut1_for	TTTAAACTGAACAAATTGAATAAACTTAATTTTG	pBW7
opuB_mut1_rev	TTTTTCAGACAATTGAATGCTTC	
opuB_mut2_for	CAAATTGAATAAACTTAATTTTGGAG	pBW8
opuB_mut2_rev	TTAAATTTAATTTTTCAGACAATTGAATG	
opuB_mut3_for	CTGAAAAATTCCCTTTAAACTGAACAAATTGAATAAA CTTAATTTTG	pBW9
opuB_mut3_rev	ACAATTGAATGCTTCCCATTATAG	
opuB_mut4_for	CAAATTGAATAAACTTAATTTTGGAG	pBW10
opuB_mut4_rev	TTTTTCAGACAATTGAATGCTTC	
opuB_mut5_for	TTAAACTGAACAAATTGAATAAACTTAATTTTG	pBW11
opuB_mut5_rev	AAATTTAATTTTTCAGACAATTGAATG	
opuB_mut6_for	AAACTGAACAAATTGAATAAACTTAATTTTG	pBW12
opuB_mut6_rev	ATTTAATTTTTCAGACAATTGAATGC	
GbsRbind_mut1_for	TTTTATTTAACAAACTTTATTTACGTC	pDH2 1.1
GbsRbind_mut1_rev	TGTTTTTAACAACCTTAATCTAAC	
GbsRbind_mut2_for	CAAACTTAATTTACGTCAAGG	pDH2 2.1
GbsRbind_mut2_rev	AAAAATTTAATGTTTTTAACAACCTTAATC	
GbsRbind_mut3_for	CCCTTTTTATTTAACAAACTTTATTTACGTC	pDH2 3.1
GbsRbind_mut3_rev	AATGTTTTTAACAACCTTAATCTAAC	
GbsRbind_mut2_for	CAAACTTAATTTACGTCAAGG	pDH2 5.2
GbsRbind_mut1_rev	TGTTTTTAACAACCTTAATCTAAC	
GbsRbind_mut6_for	TTTTATTTAACAAACTTTATTTACGTCAAG	pDH2 6.5
GbsRbind_mut6_rev	AAATTTAATGTTTTTAACAACCTTAATC	
GbsRbind_mut7_for	ATTTAACAAACTTTATTTACGTCAAG	pDH2 7.p
GbsRbind_mut6_rev	AAATTTAATGTTTTTAACAACCTTAATC	
gbsAB_zu_opuC_1_for	GTTAAAAACATTAAATTTTTATTTAACAAAGTTTATTT ACGTCAAGGAGGCTTATATGAG	pARO14
gbsAB_zu_opuC_1_rev	CTCATATAAGCCTCCTTGACGTAAATAAATAAACTTTG TTAAATAAAATTTAATGTTTTTAAC	

Table S4. Oligonucleotides used in this study (continuation).

Primer name	Primer sequence (5´-3´)[a)]	Resulting plasmid / application
gbsAB_zu_opuC_kompl_for	GTTAAAAACATTAAATTTTTATTTAACAAAGTTC AAATACGTCAAGGAGGCTTATATGAG	pARO15
gbsAB_zu_opuC_kompl_rev	CTCATATAAGCCTCCTTGACGTATTTGAACTTTG TTAAATAAAAATTTAATGTTTTTAAC	
gbsAB_zu_opuC_2_for	GTAAAAACATTAAATTTTTATTTAACAAACTTCA TTTACGTCAAGGAGGCTTATATGAG	pTM22
gbsAB_zu_opuC_2_rev	CTCATATAAGCCTCCTTGACGTAAATGAAGTTTG TTAAATAAAAATTTAATGTTTTTAAC	
gbsAB_zu_opuC_4_for	GTTAAAAACATTAAATTTTTATTTAACAAACTTT ATATACGTCAAGGAGGCTTATATGAG	pTM23
gbsAB_zu_opuC_4_rev	CTCATATAAGCCTCCTTGACGTATATAAAGTTTG TTAAATAAAAATTTAAGTTTTTAAC	
gbsAB_zu_opuC_3_for	GTTAAAAACATTAAATTTTTATTTAACAAACTTT AATTACGTCAGGAGGCTTATATGAG	pTM24
gbsAB_zu_opuC_3_rev	CTCATATAAGCCTCCTTGACGTAATTAAAGTTTG TTAAAATAAAAATTTAATGTTTTTAAC	
GbsR Knout Spc P1	TCTAAATCCGCGTCCTTGAAAACAATATTT	*Δ(gbsR::spc)*
GbsR Knout Spc P2	CTTGCCAGTCACGTTACGTTATTAGTTATATATA GCTGCAAATTCTGGATTTTCATCCAT	
GbsR Knout Spc P3	TCATAGCTGTTTCCTGTGTGAAATTGTTATAGAC CGGAGAAATTTTTAAATTTATACCGG	
GbsR Knout Spc P4	TTAAGCGGTAAAAGAGACTGTATGAAATTG	
GbsR Knout Spc P5	ATGGATGAAAATCCAGAATTTGCAGCTATATAT AACTAATAACGTAACGTGACTGGCAAG	
GbsR Knout Spc P6	CCGGTATAAATTTAAAAATTTCTCCGGTCTATAA CAATTTCACACAGGAAACAGCTATGA	
OpcR Knout P1	ATAAATTCTTCAACAAACTCATTTGCCGG	*Δ(opcR::zeo)*
OpcR Knout Zeo P2	CCATATCAAGATAACTTCGTATAATGTATGTTGA AGGCATTCCAAACGTATGCATATTTT	
OpcR Knout Zeo P3	CCATATCAAGATAACTTCGTATAATGTATGTTGA AGGCATTCCAAACGTATGCATATTTT	
OpcR Knout P4	GTAAAGCAATACTCGTCTGCTTTTGTTTTA	
OpcR Knout Zeo P5	AAAATATGCATACGTTTGGAATGCCTTCAACAT ACATTATACGAAGTTATCTTGATATGG	
OpcR Knout Zeo P6	TATTTTAGAGAGCTGCATTCTTTTGTTTTCTAAT GTATGCTATACGAAGTTATTCAGTCC	
YvaV Knout P1	GATAAATTCCTCAACAAATTCGTCTGCC	*Δ(yvaV::tet)*
Yvav Knout Tet P2	CCGTAATGCTATGTTAGCATTACTCTTTTCCATG TTTTCCGCGATTCTTTCTATAAAATG	
YvaV Kneu2 Tet P3	AAATTGTTATCCGCTCACAATTCCACACAACATA TTTGAGAGCGAAGACATTTTTAAATATGTG	
YvaV Knout P4	CAGTGAAATAAACCGGTAAATCTAGGTCTC	
YvaV Knout Tet P5	CATTTTATAGAAAGAATCGCGGAAAACATGGAA AAGAGTAATGCTAACATAGCATTACGG	
YvaV Knout2 Tet P6	CACATATTTAAAAATGTCTTCGCTCTCAAATATG TTGTGTGGAATTGTGAGCGGATAACAATTT	
BS_gbsA_for	GGGACTTTGACAGTTTAAAAACC	EMSA
BS_gbsA_rev_Dy781	DY781-ATAAGCCTCCTTGACGTAAATAA	

Table S5. Plasmids used in this study.

Plasmid	Description	Resistance	Reference
pASG-IBA3	Expression plasmid for *E. coli* with a AHT-inducible *tet*-promoter and a C-terminal *Strep*-tag II	*bla*	IBA (Göttingen, Germany)
pJMB1[a)]	(*amyE::treA*) *cat*	*bla, cat*	(Hoffmann et al., 2013)
p7Z6	Zeocin resistance cassette (*zeo*)	*bla, zeo*	(Yan et al., 2008)
pDG1515	Tetracycline resistance cassette (*tet*)	*bla, tet*	(Guerout-Fleury et al., 1995)
pDG1726	Spectinomycin resistance cassette (*spc*)	*bla, spc*	(Guerout-Fleury et al., 1995)
pDH2[b)]	*amyE::*Φ[*gbsA´-treA*)1] *cat*	*bla, cat*	(Nau-Wagner et al., 2012)
pARO14	Substitution of CTTTATT/GTTTATT within the predicted GbsR-BS [Φ(*gbsA´-treA*)8]	*bla, cat*	This study
pARO15	Substitution of CTTTATT/GTTCAAA within the predicted GbsR-BS [Φ(*gbsA´-treA*)9]	*bla, cat*	This study
pBW7	Deletion of TTAAA within the GbsR-BS [Φ(*opuB´-treA*)2]	*bla, cat*	This study
pBW8	Deletion of ACTGAA within the GbsR-BS [Φ(*opuB´-treA*)3]	*bla, cat*	This study
pBW9	Substitution of TTAAAT/TTCCCT within the GbsR-BS [Φ(*opuB´-treA*)6]	*bla, cat*	This study
pBW10	Deletion of TTAAATTTAAACTGAA within the GbsR-BS [Φ(*opuB´-treA*)4]	*bla, cat*	This study
pBW11	Insertion of TTAAATTTAAACTGAA/TTAAATTTT TAAACTGAA within the GbsR-BS [Φ(*opuB´-treA*)5]	*bla, cat*	This study
pBW12	Deletion of TTAAATTTAAACTGAA/TTAAATAAA CTGAA within the GbsR-BS [Φ(*opuB´-treA*)7]	*bla, cat*	This study
pBW34	*amyE::*Φ(*opcR-treA*)	*bla, cat*	This study
pGNB10	*amyE::*Φ[*gbsR´-treA*)1] *cat*	*bla, cat*	This study

a) In this plasmid, a promoter-less *treA* reporter gene is flanked by 5’- and 3’-segments of the *B. subtilis amyE* gene, thereby allowing the stable integration of the construct as a single copy into the chromosome via a double homologous recombination event. Integration of the construct can be selected for via a chloramphenicol resistance mediated by the *cat* gene that is present behind the 3’-end of the *treA* reporter gene. The double homologous recombination event disrupts the non-essential *amyE* gene, thereby resulting in an amylase-minus phenotype that can be scored on starch plated flooded with an iodine solution.

b) All operon fusion constructs in which a promoter region of interest is fused to a promoter-less *treA* gene can be integrated as a single copy reporter fusion into the chromosomal *B. subtilis amyE* gene.

Table S5. Plasmids used in this study (continuation).

Plasmid	Description	Resistance	Reference
pDH2 1.1	Deletion of TTAAAT within the GbsR-BS [Φ(*gbsA´-treA*)2]	*bla, cat*	This study
pDH2 2.1	Deletion of ATTTAA within the GbsR-BS [Φ(*gbsA´-treA*)3]	*bla, cat*	This study
pDH2 3.1	Substitution of TTAAAT/TTCCCT within the GbsR-BS [Φ(*gbsA´-treA*)4]	*bla, cat*	This study
pDH2 5.2	Deletion of TTAAATTTTTATTTAA within the GbsR-BS [Φ(*gbsA´-treA*)5]	*bla, cat*	This study
pDH2 6.5	Insertion of TTAAATTTTTATTTAA/TTAAATTTTTT TATTTAA within the GbsR-BS [Φ(*gbsA´-treA*)6]	*bla, cat*	This study
pDH2 7.p	Deletion of TTAAATTTTTATTTAA / TTAAATTTATTTAA within the GbsR-BS [Φ(*gbsA´-treA*)7]	*bla, cat*	This study
pSTH02	*B. subtilis gbsR* gene cloned into pASK-IBA3	*bla*	This study
pSTH62	*amyE::*Φ(*opuCA-treA*) *cat*	*bla, cat*	This study
pSTH67	*amyE::*Φ(*opuBA-treA*) *cat*	*bla, cat*	This study
pTM22	Substitution of CTTTATT/CTTCATT within the predicted GbsR-BS [Φ(*gbsA´-treA*)10]	*bla, cat*	This study
pTM23	Substitution of CTTTATT/CTTTATA within the predicted GbsR-BS [Φ(*gbsA´-treA*)12]	*bla, cat*	This study
pTM24	Substitution of CTTTATT/CTTTAAT within the predicted GbsR-BS [Φ(*gbsA´-treA*)11]	*bla, cat*	This study

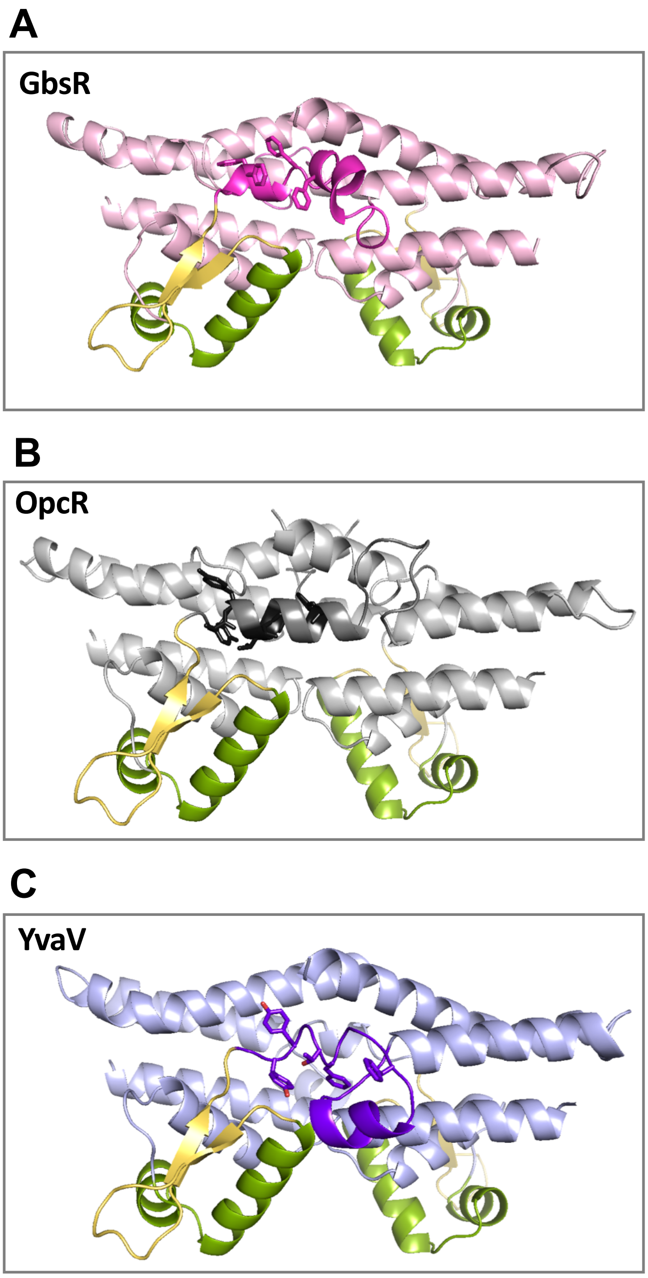

Fig. S1. Putative three-dimensional structures of GbsR and its homologues OpcR and YvaV of *B. subtilis*. *In silico* models of the GbsR (A), OpcR (B) and YvaV proteins (C) were built using the protein structure homology server "swiss model" (Waterhouse et al., 2018) with the crystal structure of the DNA-binding protein Mj223 of *M. jannaschii* (PDB entry 1KU9) (Ray et al., 2003) as the template. The winged helix-turn-helix region is represented in green, the inter-domain linker region is shown in yellow. The variably folded region covering the aromatic residues potentially involved in effector-binding are highlighted in pink (A), dark grey (B) and dark blue (C).

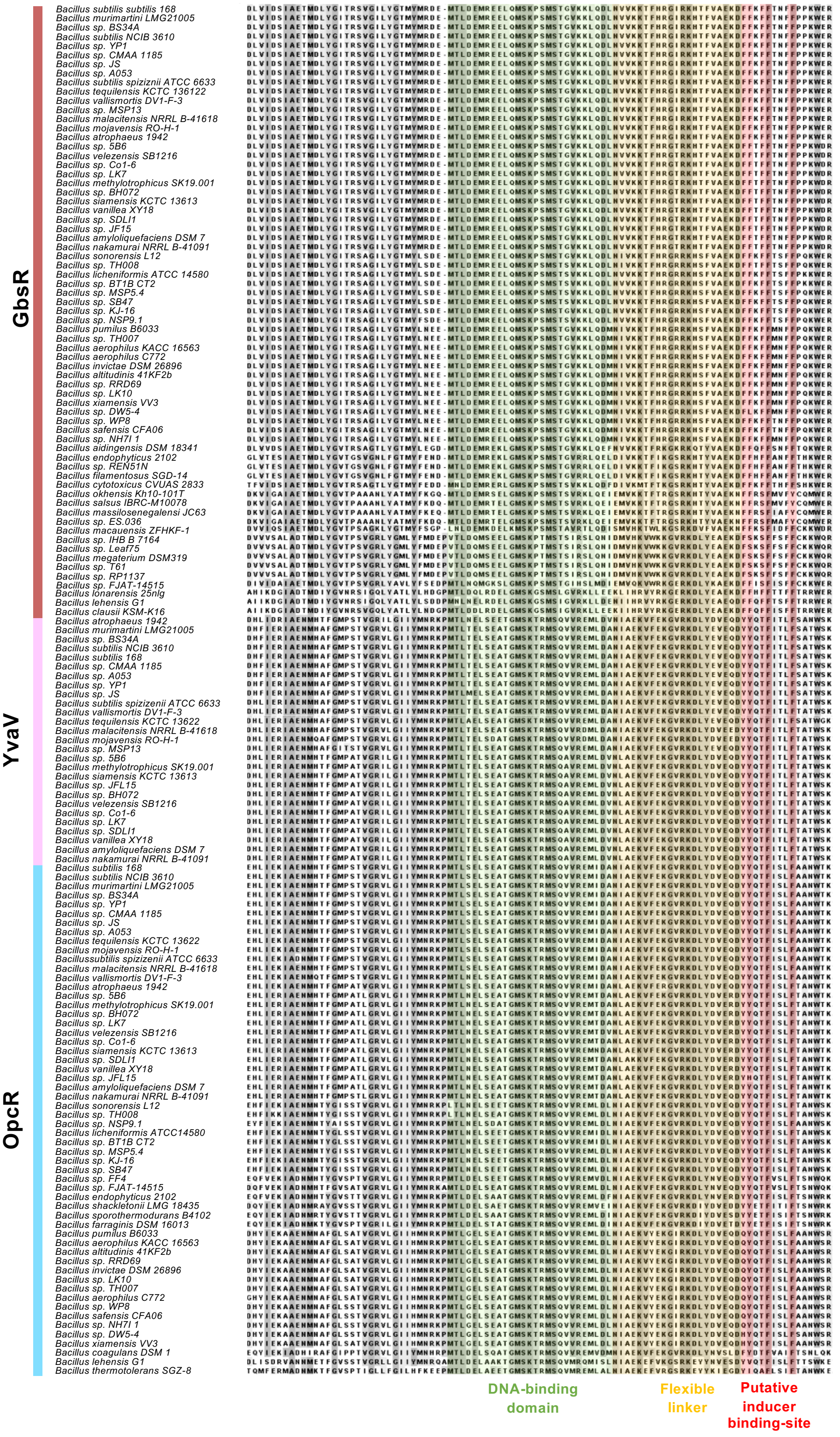

Fig. S2. Amino acid sequence alignment of GbsR-like proteins from various members of the genus *Bacillus*. The amino acid sequences of 68 GbsR-type proteins (Nau-Wagner *et al.*, 2012), 35 OpuAR-type proteins (Ronzheimer et al., 2018), 56 OpcR-type proteins (Lee et al., 2013), and 27 YvaV-type proteins were aligned using the MAFFT web server (Katoh et al., 2017). Only the N-terminal domain of the proteins is depicted. The amino acids of the winged helix-turn-helix DNA-binding motive, the flexible inter-domain linker, and the suggested inducer binding-site (Nau-Wagner et al., 2012; Ronzheimer et al., 2018) are marked. Highly conserved amino acids are shaded in grey.

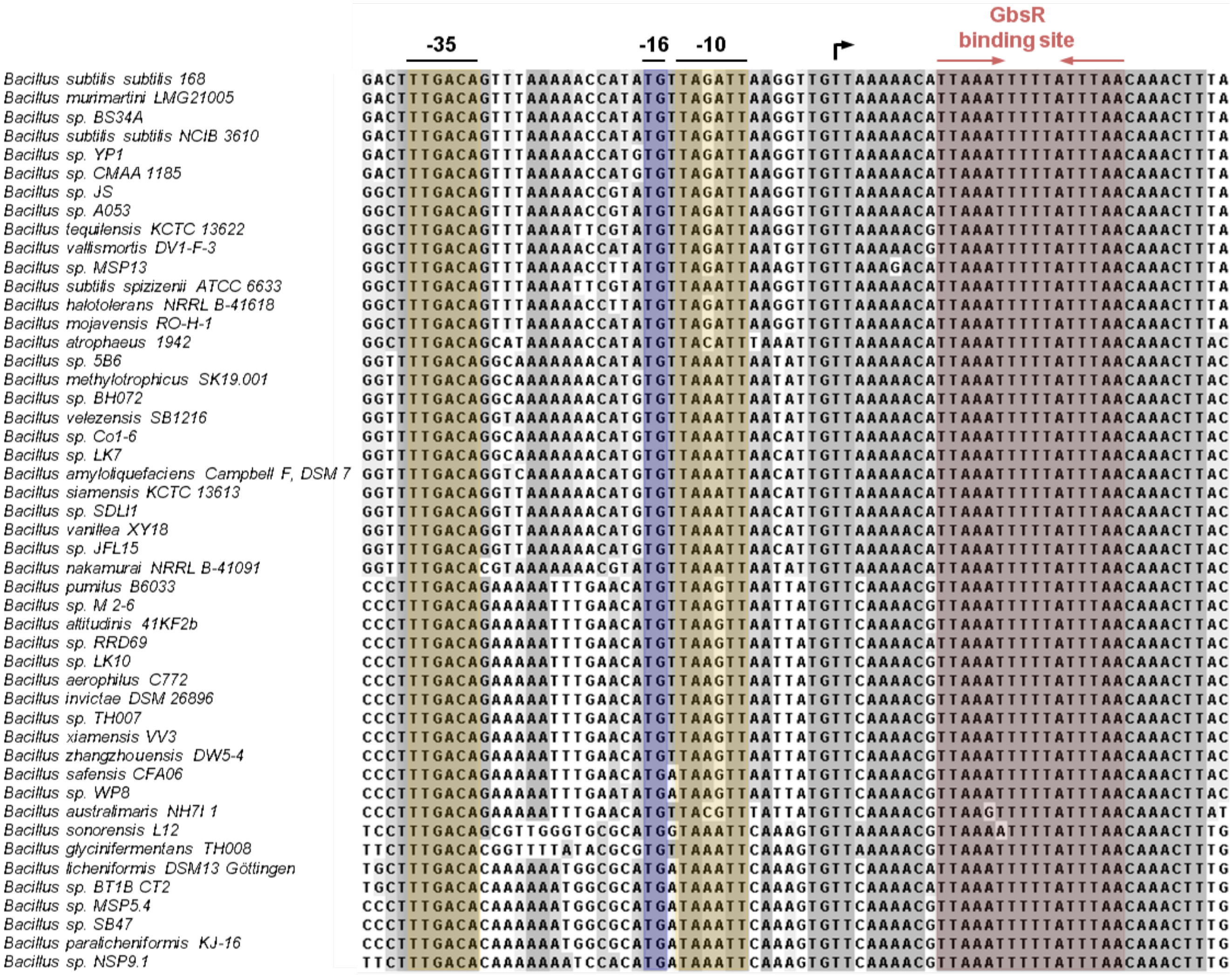

Figure S3. *In silico* analysis of the putative GbsR binding site of the *gbsAB* operon among members of the genus *Bacillus*. An alignment of the DNA-sequences of the regulatory regions of the various *gbsAB* gene clusters is shown. The -35, -16 and -10 sequence of the SigA-type promoter, the transcriptional start site (indicated by a bent arrow) (Boch et al., 1996) and the ribosomal binding site (RBS) are highlighted. Red arrows highlight the GbsR binding site.

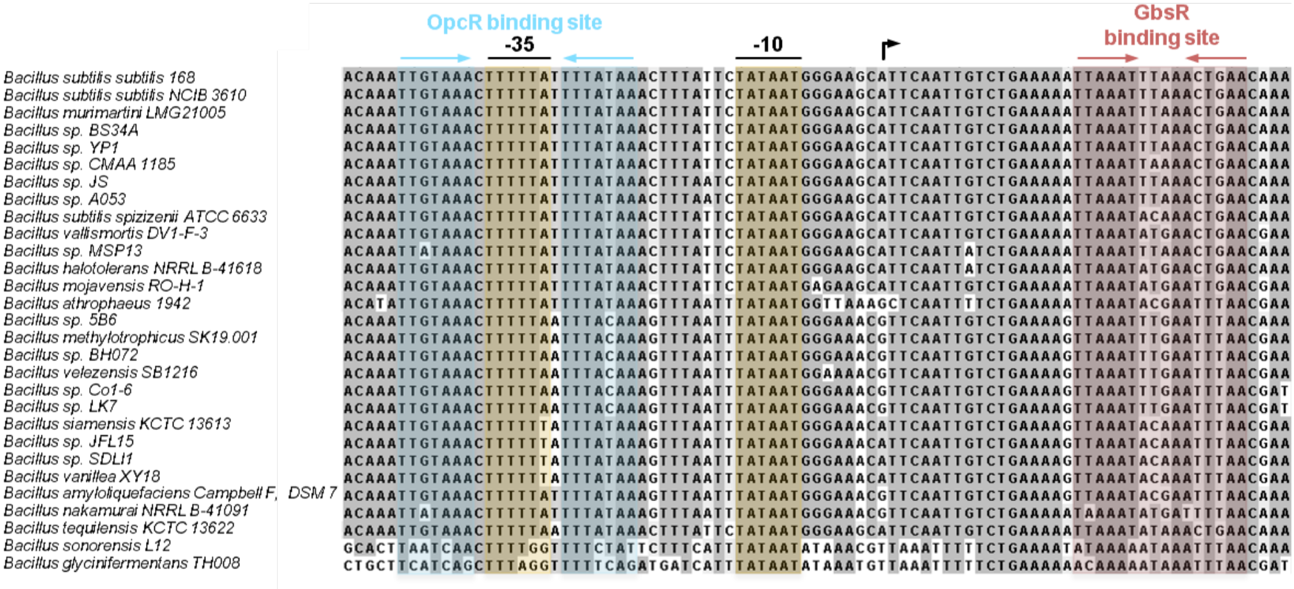

Figure S4. *In silico* analysis of the putative GbsR and OpcR binding sites of the *opuB* operon among the genus *Bacillus*. An alignment of the DNA-sequences of the regulatory regions of *opuB* gene clusters of various Bacilli is shown. The -35 and -10 sequence of the SigA-type promoter, the transcriptional start site (indicated by a bent arrow) (Kappes et al., 1999) and the ribosomal binding site (RBS) are highlighted. Red arrows highlight the suggested GbsR binding site. Blue arrows highlight the suggested OpcR binding site (Lee *et al*., 2013).

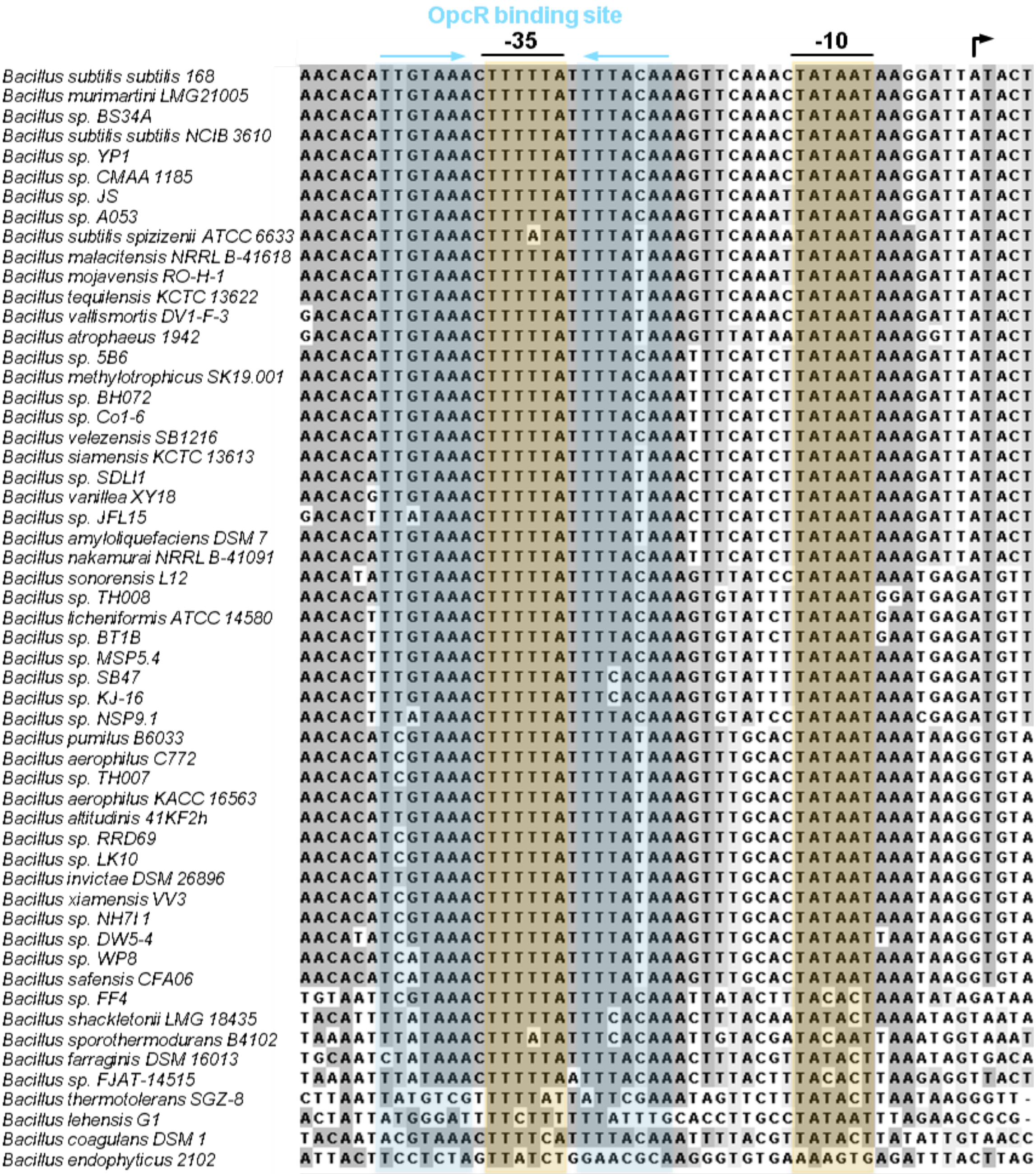

Figure S5. *In silico* analysis of the OpcR binding site of the *opuC* operon among members of the genus *Bacillus*. Alignment of the DNA-sequences of the regulatory regions from the various *opuC* gene cluster that contain an adjacent *opcR* gene. The -35 and -10 sequence of the predicted SigA-type promoter, the transcriptional start site (indicated by a bent arrow) (Kappes et al., 1999) and the ribosomal binding site (RBS) are highlighted. Blue arrows highlight the suggested OpcR binding site (Lee *et al.*, 2013).

Condition	Melting point [°C]
As isolated	n.d
0.2 M L-Arginine, L-Glutamic acid	63
0.4 M Glycine	75
0.4 M L-Proline	69
0.4 M β-Alanine	65
0.4 M L-Serine	77
0.4 M L-Argininamide dihydrochloride	53
0.4 M 6-Aminohexanoic acid	53
0.4 M Gly-gly	77
0.16 M Gly-gly-gly	77
2 M Betaine monohydrate	75
0.6 M D-(+)-Trehalose dihydrate	77
1.6 M Xylitol	77
1.6 M D-Sorbitol	79
1.6 M Sucrose	83
0.4 M Hydroxyectoine	68; 79; 83; 85
2 M Trimethylamine N-oxide dihydrate	72
0.4 M Spermine tetrahydrochloride	62
0.4 M 5-Aminovaleric acid	62
0.04 M Ethylenediaminetetraacetic acid disodium salt dihydrate	73
0.8 M Non Detergent Sulfobetaine 221 (NDSB-221)	76
0.4 M Taurine	76
0.4 M Acetamide	73
0.4 M Oxalic acid dihydrate	77
0.4 M Succinic acid (pH 7)	69
4 % (v/v) Tacsimate (pH 7)	70
20% (v/v) Tetraethylammonium bromide	76
20% (v/v) Cholin acetate	71
20% (v/v) 1-Ethyl-3-methylimidazolium acetate	71
20% (v/v) 1-Butyl-3-methylimidazolium chloride	71
20% (v/v) Ethylammonium nitrate	70
0.4 M Ammonium sulfate	73
0.2 M Gadolinium(III) chloride hexahydrate	72
0.2 M Cesium chloride	73
0.2 M 4-Aminobutyric acid (GABA)	71
0.4 M Lithium nitrate	77
0.4 M DL-Malic acid (pH 7)	65
0.4 M Lithium citrate tribasic tetrahydrate	61
0.2 M Ammonium acetate	64
0.2 M Sodium benzenesulfonate	72
0.2 M Sodium p-toluenesulfonate	77
0.8 M Sodium chloride	67
1.12 M Potassium chloride	64
1.12 M Lithium chloride	59
0.8 M Sodium bromide	67
32% (v/v) Glycerol	68
40% (v/v) Glycerol	54; 80
8% (v/v) Ethylene glycol	62
8% (v/v) Polyethylene glycol 200	57
4% (v/v) Polyethylene glycol monomethyl ether 750	51
40% (v/v) Formamide	68
8% (v/v) Polyethylene glycol 400	55
20% (v/v) Pentaerythritol ethoxylate (15/4 EO/OH)	65
8% (v/v) 1,2-Propanediol	49
2.4% (w/v) Polyethylene glycol monomethyl ether 1,900	37
2.4% (w/v) Polyethylene glycol 3,350	59
2.4% (w/v) Polyethylene glycol 8,000	71
1.6% (w/v) Polyvinylpyrrolidone K15	49
1.6% (w/v) Polyethylene glycol 20,000	56
0.008 M (2-Hydroxypropyl)-β-cyclodextrin	67
0.064 M α-Cyclodextrin	68
0.04 M Methyl-β-cyclodextrin	73
0.16 M Succinic acid (pH 5.5)	65
0.16 M MES monohydrate (pH 6)	72
0.16 M BIS-TRIS (pH 6.5)	66
0.16 M Imidazole (pH 7)	60
0.16 M HEPES (pH 7.5)	54
0.18 M Tris (pH 8)	54
0.16 M BIS-TRIS Propane (pH 8.5)	50
0.16 M AMPD (pH 9)	48
0.16 M Glycine (pH 9.5)	42
0.19 M NaCl	60
0.16 M Sodium citrate tribasic dihydrate (pH 5); 0.19 M NaCl	59
0.16 M Succinic acid (pH 5.5); 0.19 M NaCl	61
0.16 M MES monohydrate (pH 6); 0.19 M NaCl	63
0.16 M BIS-TRIS (pH 6.5); 0.19 M NaCl	59
0.16 M Imidazole (pH 7); 0.19 M NaCl	55
0.18 M Tris (pH 8); 0.19 M NaCl	54
0.16 M BIS-TRIS Propane (pH 8.5); 0.19 M NaCl	54
0.16 M AMPD (pH 9); 0.19 M NaCl	52
0.35 M NaCl	58
0.16 M Sodium citrate tribasic dihydrate (pH 5); 0.35 M NaCl	60
0.16 M Succinic acid (pH 5.5); 0.35 M NaCl	61
0.16 M MES monohydrate (pH 6); 0.35 M NaCl	60
0.16 M BIS-TRIS (pH 6.5); 0.35 M NaCl	59
0.16 M Imidazole (pH 7); 0.35 M NaCl	57
0.16 M HEPES (pH 7.5); 0.35 M NaCl	59
0.18 M Tris (pH 8); 0.35 M NaCl	59
0.16 M BIS-TRIS Propane (pH 8.5); 0.35 M NaCl	59
0.16 M AMPD (pH 9); 0.35 M NaCl	57
0.16 M Glycine (pH 9.5); 0.35 M NaCl	82; 85
0.51 M NaCl	60
0.16 M Sodium citrate tribasic dihydrate (pH 5); 0.51 M NaCl	60
0.16 M Succinic acid (pH 5.5); 0.51 M NaCl	62
0.16 M MES monohydrate (pH 6); 0.51 M NaCl	61
0.16 M BIS-TRIS (pH 6.5); 0.51 M NaCl	61
0.16 M Imidazole (pH 7); 0.51 M NaCl	60
0.18 M Tris (pH 8); 0.51 M NaCl	61
0.16 M BIS-TRIS Propane (pH 8.5); 0.51 M NaCl	61; 77
0.16 M AMPD (pH 9); 0.51 M NaCl	60
0.16 M Glycine (pH 9.5); 0.51 M NaCl	61
0.67 M NaCl	63
0.16 M Sodium citrate tribasic dihydrate (pH 5); 0.67 M NaCl	62
0.16 M Succinic acid (pH 5.5); 0.67 M NaCl	65
0.16 M MES monohydrate (pH 6); 0.67 M NaCl	63
0.16 M BIS-TRIS (pH 6.5); 0.67 M NaCl	64
0.16 M Imidazole (pH 7); 0.67 M NaCl	62
0.16 M HEPES (pH 7.5); 0.67 M NaCl	64
0.18 M Tris (pH 8); 0.67 M NaCl	64
0.16 M BIS-TRIS Propane (pH 8.5); 0.67 M NaCl	64
0.16 M AMPD (pH 9); 0.67 M NaCl	63
0.16 M Glycine (pH 9.5); 0.67 M NaCl	64
0.83 M NaCl	64
0.16 M Sodium citrate tribasic dihydrate (pH 5); 0.83 M NaCl	63
0.16 M Succinic acid (pH 5.5); 0.83 M NaCl	66
0.16 M MES monohydrate (pH 6); 0.83 M NaCl	65
0.16 M BIS-TRIS (pH 6.5); 0.83 M NaCl	66
0.16 M Imidazole (pH 7); 0.83 M NaCl	64
0.16 M HEPES (pH 7.5); 0.83 M NaCl	67
0.18 M Tris (pH 8); 0.83 M NaCl	65
0.16 M BIS-TRIS Propane (pH 8.5); 0.83 M NaCl	65
0.16 M AMPD (pH 9); 0.83 M NaCl	64
0.16 M Glycine (pH 9.5); 0.83 M NaCl	66
1.63 M NaCl	71
0.16 M Sodium citrate tribasic dihydrate (pH 5); 1.63 M NaCl	67
0.16 M Succinic acid (pH 5.5); 1.63 M NaCl	70
0.16 M MES monohydrate (pH 6); 1.63 M NaCl	70
0.16 M BIS-TRIS (pH 6.5); 1.63 M NaCl	71
0.16 M Imidazole (pH 7); 1.63 M NaCl	70
0.16 M HEPES (pH 7.5); 1.63 M NaCl	72
0.18 M Tris (pH 8); 1.63 M NaCl	72
0.16 M BIS-TRIS Propane (pH 8.5); 1.63M NaCl	71
0.16 M AMPD (pH 9); 1.63 M NaCl	71
0.16 M Glycine (pH 9.5); 1.63 M NaCl	71
3.23 M NaCl	74
0.16 M Sodium citrate tribasic dihydrate (pH 5); 3.23 M NaCl	67
0.16 M Succinic acid (pH 5.5); 3.23 M NaCl	72
0.16 M MES monohydrate (pH 6); 3.23 M NaCl	71
0.16 M BIS-TRIS (pH 6.5); 3.23 M NaCl	74
0.16 M Imidazole (pH 7); 3.23 M NaCl	74
0.16 M HEPES (pH 7.5); 3.23 M NaCl	77
0.18 M Tris (pH 8); 3.23 M NaCl	77
0.16 M BIS-TRIS Propane (pH 8.5); 3.23 M NaCl	77
0.16 M AMPD (pH 9); 3.23 M NaCl	76
0.16 M Glycine (pH 9.5); 3.23 M NaCl	76

Figure S6. Buffer-screen to improve the stability of the purified GbsR-*Strep*-tag II protein. The recombinant GbsR protein was affinity purified in a 100 mM Tris-HCl (pH 7.5) and 150 mM NaCl buffer (Nau-Wagner et al., 2012). Immediately after purification, the melting points (T_m) of the GbsR protein were determined by a nanoDSF approach using a Prometheus NT.48 (NanoTemper Technologies GmbH, Munich, Germany). For these screens, 5 µl of the GbsR-*Strep*-tag II protein (concentration of 40 – 45 µM in 100 mM Tris-HCl pH 7.5, 150 mM NaCl) was mixed with 20 µl of the various buffer solutions (Solubility & Stability Screen and Solubility & Stability Screen 2 from Hampton Research, Aisa Viejo, CA, USA). The denaturation of the GbsR-*Strep*-tag II protein was followed in a temperature range between 20 ° C to 95 ° C applying a linear temperature increase of 2 ° C min^{-1}. The quantities derived from the purification buffer were included into the calculation in case of Tris (20 mM derived from the purification buffer) and NaCl (30 mM derived from the purification buffer). n.d: not detected

References

Boch, J., Kempf, B., Schmid, R., Bremer, E. (1996). Synthesis of the osmoprotectant glycine betaine in *Bacillus subtilis*: characterization of the *gbsAB* genes. *J. Bacteriol.* 178, 5121-5129. doi:10.1128/jb.178.17.5121-5129.1996

Guerout-Fleury, A.M., Shazand, K., Frandsen, N., Stragier, P. (1995). Antibiotic-resistance cassettes for *Bacillus subtilis*. *Gene* 167, 335-336. doi:10.1016/0378-1119(95)00652-4

Hoffmann, T., Wensing, A., Brosius, M., Steil, L., Völker, U., Bremer, E. (2013). Osmotic control of opuA expression in Bacillus subtilis and its modulation in response to intracellular glycine betaine and proline pools. J. Bacteriol. 195, 510-522. doi:10.1128/jb.01505-12

Kappes, R.M., Kempf, B., Kneip, S., Boch, J., Gade, J., Meier-Wagner, J., Bremer, E. (1999). Two evolutionarily closely related ABC transporters mediate the uptake of choline for synthesis of the osmoprotectant glycine betaine in *Bacillus subtilis*. *Mol. Microbiol.* 32, 203-216. doi: 10.1046/j.1365-2958.1999.01354.x

Katoh, K., Rozewicki, J., Yamada, K.D. (2017). MAFFT online service: multiple sequence alignment, interactive sequence choice and visualization. Brief. Bioinform. 1-7. doi:10.1093/bib/bbx108

Lee, C.H., Wu, T.Y., Shaw, G.C. (2013). Involvement of OpcR, a GbsR-type transcriptional regulator, in negative regulation of two evolutionarily closely related choline uptake genes in *Bacillus subtilis*. *Microbiology* 159, 2087-2096. doi: 10.1099/mic.0.067074-0

Nau-Wagner, G., Opper, D., Rolbetzki, A., Boch, J., Kempf, B., Hoffmann, T., Bremer, E. (2012). Genetic control of osmoadaptive glycine betaine synthesis in Bacillus subtilis through the choline-sensing and glycine betaine-responsive GbsR repressor. J. Bacteriol. 194, 2703-2714. doi:10.1128/jb.06642-11

Ray S. S., Bonanno J. B., Chen H., de Lencastre H., Wu S., Tomasz A., et al. (2003). X-ray structure of an M. jannaschii DNA-binding protein: implications for antibiotic resistance in S. aureus. Proteins 50, 170–173. doi:org/10.1002/prot.10272

Ronzheimer, S., Warmbold, B., Arnhold, C., Bremer, E. (2018). The GbsR family of transcriptional regulators: functional characterization of the OpuAR repressor. Front. Microbiol. 9, 2536. doi:10.3389/fmicb.2018.02536

Smith, J.L., Goldberg, J.M., and Grossman, A.D. (2014). Complete genome sequences of Bacillus subtilis subsp. subtilis laboratory strains JH642 (AG174) and AG1839. Genome Announc. 2. doi:10.1128/genomea.00663-14

Teichmann, L., Chen, C., Hoffmann, T., Smits, S.H.J., Schmitt, L., Bremer, E. (2017). From substrate specificity to promiscuity: hybrid ABC transporters for osmoprotectants. Mol. Microbiol. 104, 761-780. doi:10.1111/mmi.13660

Waterhouse, A., Bertoni, B., Bienert, S., Studer, G., Tauriello, G., Gumienny, R., et al. (2018). SWISS-MODEL: homology modelling of protein structures and complexes, Nucl. Ac. Res., 46, W296–W303. doi:10.1093/nar/gky427

Yan, X., Yu, H.J., Hong, Q., Li, S.P. (2008). Cre/lox system and PCR-based genome engineering in Bacillus subtilis. Appl. Environ. Microbiol. 74, 5556-5562. doi:10.1128/aem.01156-08
